# Supplementary material for: Mixed alkali-ion transport and storage in atomic-disordered honeycomb layered NaKNi2TeO6
Source: Nat Commun. 2021 Aug 2;12:4660. doi: 10.1038/s41467-021-24694-5 (PMC8329229; doi:10.1038/s41467-021-24694-5)
Supplement: Supplementary file 1 — Supplementary Information [file 41467_2021_24694_MOESM1_ESM.pdf]

Supplementary Information

Mixed Alkali-Ion Transport and Storage In Atomic-Disordered Honeycomb Layered $NaKNi_2TeO_6$

Titus Masese[1,2]*, Yoshinobu Miyazaki[3]*, Josef Rizell[1,4], Godwill Mbiti Kanyolo[5]*, Chih-Yao Chen[2], Hiroki Ubukata[6], Keigo Kubota[2], Kartik Sau[7], Tamio Ikeshoji[7], Zhen-Dong Huang[8*], Kazuki Yoshii[1], Teruo Takahashi[3], Miyu Ito[3], Hiroshi Senoh[1], Jinkwang Hwang[9], Abbas Alshehabi[10], Kazuhiko Matsumoto[2,9], Toshiyuki Matsunaga[11], Kotaro Fujii[12], Masatomo Yashima[12], Masahiro Shikano[1], Cédric Tassel[6], Hiroshi Kageyama[6], Yoshiharu Uchimoto[11], Rika Hagiwara[2,9] & Tomohiro Saito[3]*

[1] Research Institute of Electrochemical Energy, National Institute of Advanced Industrial Science and Technology (AIST), 1–8–31 Midorigaoka, Ikeda, Osaka 563–8577, JAPAN

[2] AIST-Kyoto University Chemical Energy Materials Open Innovation Laboratory (ChEM-OIL), Sakyo–ku, Kyoto 606–8501, JAPAN

[3] Tsukuba Laboratory, Technical Solution Headquarters, Sumika Chemical Analysis Service (SCAS), Ltd.,Tsukuba, Ibaraki 300–3266, JAPAN

[4] Department of Physics, Chalmers University of Technology, SE–412 96 Göteborg, SWEDEN

[5] Department of Engineering Science, The University of Electro–Communications, 1–5–1

Chofugaoka, Chofu, Tokyo 182–8585, JAPAN

[6] Department of Energy and Hydrocarbon Chemistry, Graduate School of Engineering, Kyoto University, Nishikyo–ku, Kyoto 615–8510, JAPAN

[7] Mathematics for Advanced Materials - Open Innovation Laboratory (MathAM-OIL), National Institute of Advanced Industrial Science and Technology (AIST), c/o Advanced Institute of Material Research (AIMR), Tohoku University, Sendai 980–8577, JAPAN

[8] Key Laboratory for Organic Electronics and Information Displays and Institute of Advanced Materials (IAM), Nanjing University of Posts and Telecommunications (NUPT), Nanjing, 210023, CHINA

[9] Graduate School of Energy Science, Kyoto University, Sakyo–ku, Kyoto 606–8501, JAPAN

[10] Department of Industrial Engineering, National Institute of Technology (KOSEN), Ibaraki College, 866 Nakane, Hitachinaka, Ibaraki 312–8508 JAPAN

[11] Graduate School of Human and Environmental Studies, Kyoto University, Sakyo–ku, Kyoto 606–8501, JAPAN

[12] Department of Chemistry, School of Science, Tokyo Institute of Technology, 2–12–1–W4–17 O–okayama, Meguro–ku, Tokyo, 152–8551, JAPAN

* Correspondence and material requests should be addressed to: Titus Masese, Yoshinobu Miyazaki, Godwill Mbiti Kanyolo, Zhen-Dong Huang and Tomohiro Saito

E-mail address: titus.masese@aist.go.jp, yoshinobu.miyazaki@scas.co.jp, gmkanyolo@mail.uec.jp, iamzdhuang@njupt.edu.cn, tomohiro.saito@scas.co.jp

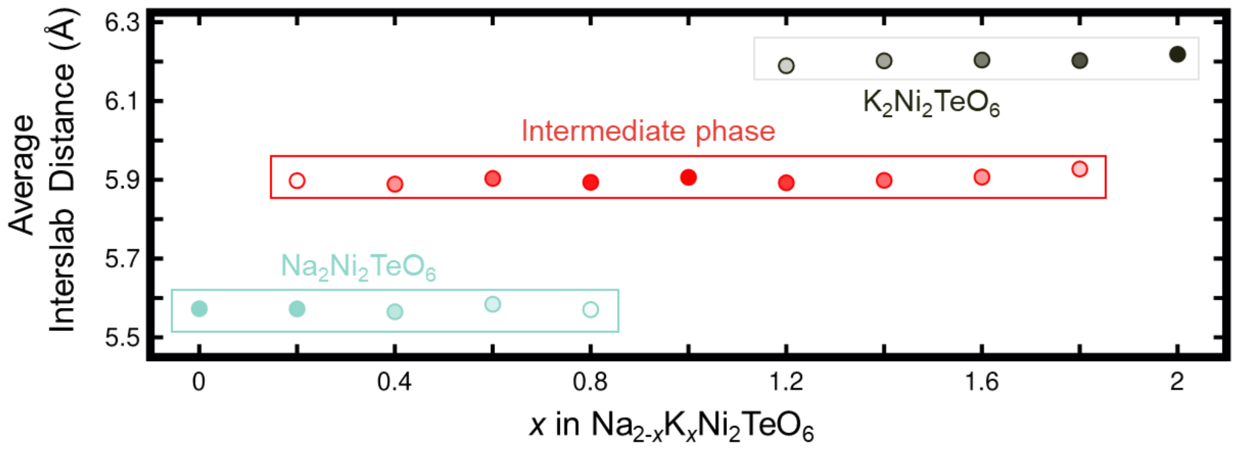

Supplementary Figure 1. Average interlayer distance (along the *c*-axis) of $Na_{2-x}K_xNi_2TeO_6$ ($0 \leq x \leq 2$), as determined by Le Bail profile fitting of the XRD patterns. All compositions, except for the end members ($Na_2Ni_2TeO_6$ and $K_2Ni_2TeO_6$) and $NaKNi_2TeO_6$, can reliably be fitted to two phases with differing average interlayer distances.

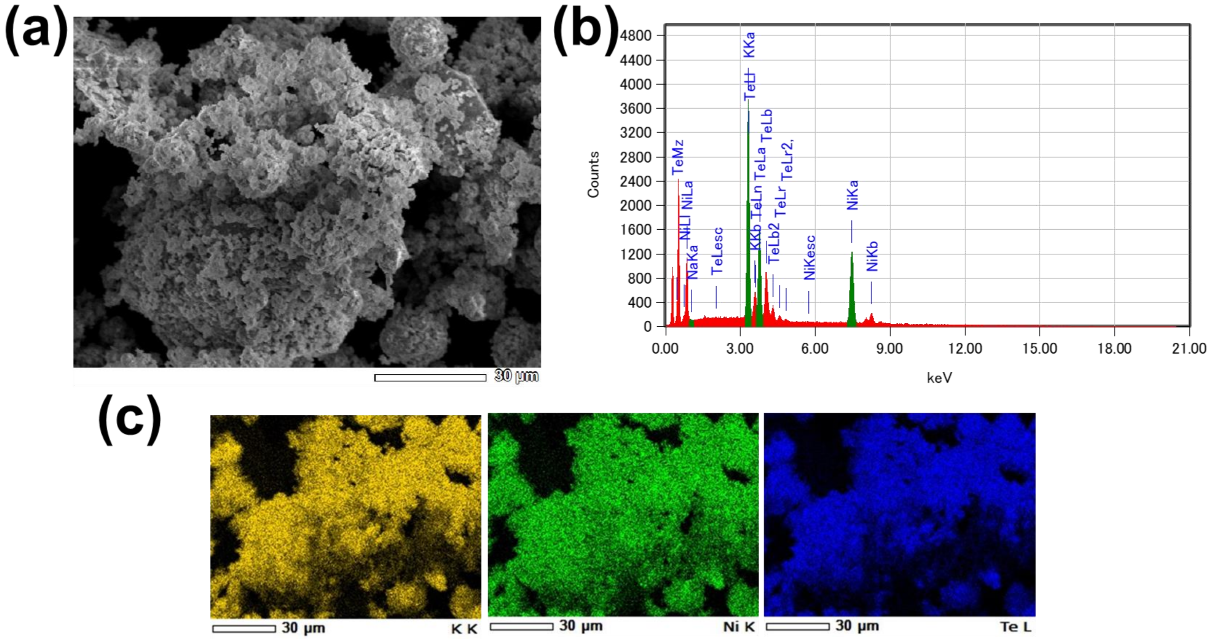

Supplementary Figure 2. (a) Scanning electron microscopy (SEM) picture of pristine $K_2Ni_2TeO_6$ powders. **(b)** SEM energy dispersive X-ray (SEM-EDX) spectrum of the powders. **(c)** Elemental mapping of $K_2Ni_2TeO_6$.

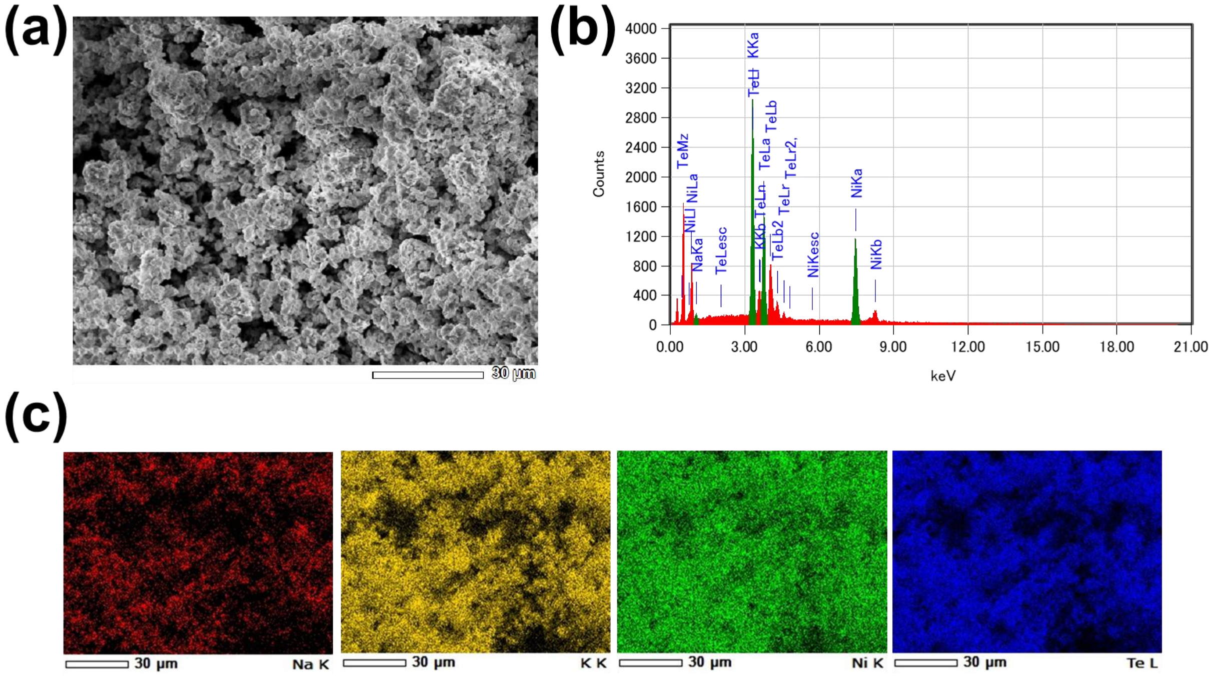

Supplementary Figure 3. (a) SEM picture of the $K_{1.8}Na_{0.2}Ni_2TeO_6$ pristine powders. **(b)** SEM-EDX spectrum of the powders. **(c)** Elemental mapping of $K_{1.8}Na_{0.2}Ni_2TeO_6$.

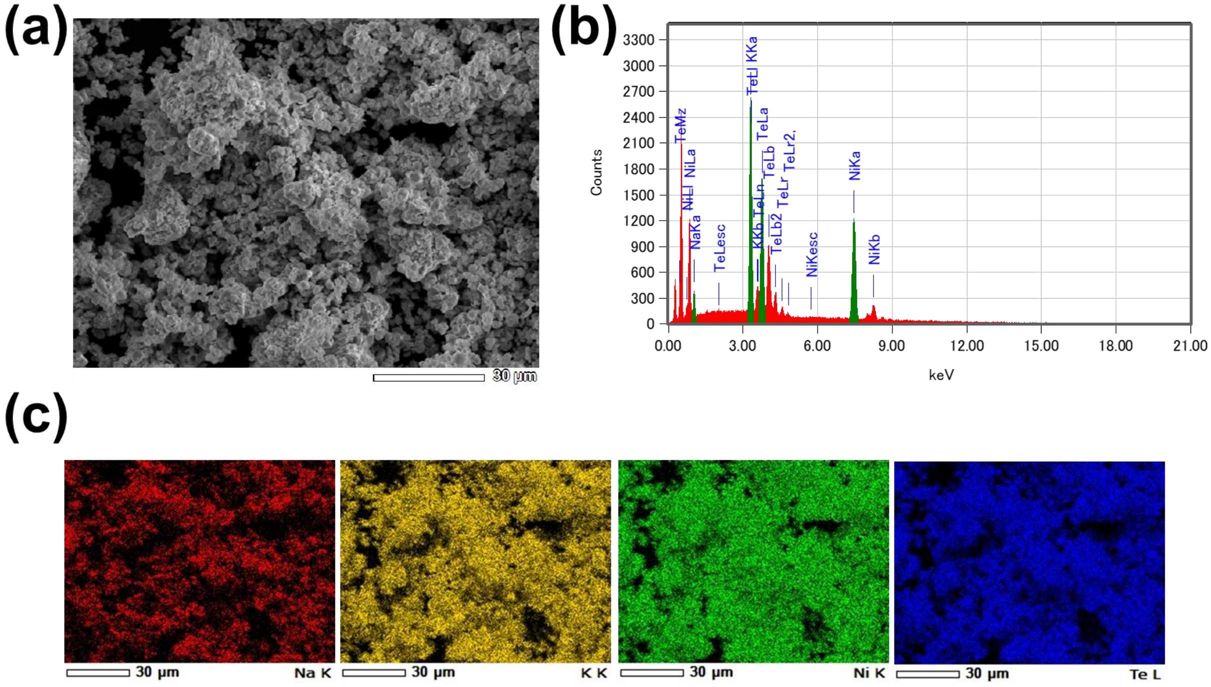

Supplementary Figure 4. **(a)** SEM picture of the $K_{1.4}Na_{0.6}Ni_2TeO_6$ powders. **(b)** SEM-EDX spectrum of the powders. **(c)** Elemental mapping of $K_{1.4}Na_{0.6}Ni_2TeO_6$.

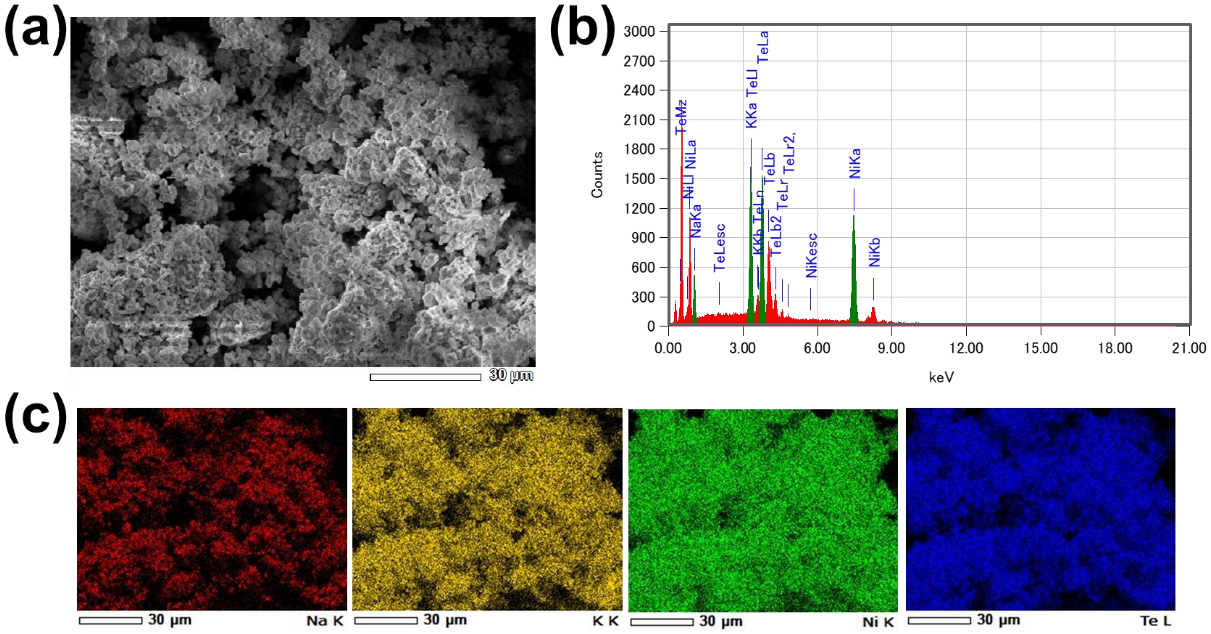

Supplementary Figure 5. (a) SEM picture of the $NaKNi_2TeO_6$ powders. **(b)** SEM-EDX spectrum of the powders. **(c)** Elemental mapping of $NaKNi_2TeO_6$.

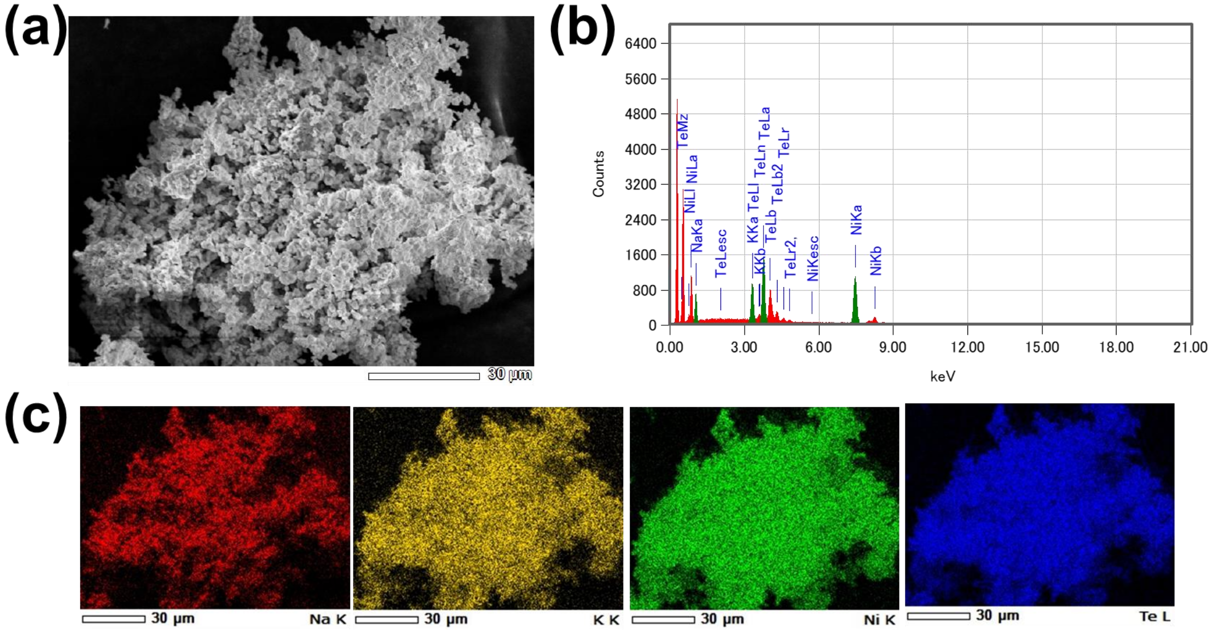

Supplementary Figure 6. (a) SEM picture of the $Na_{1.4}K_{0.6}Ni_2TeO_6$ powders. **(b)** SEM-EDX spectrum of the powders. **(c)** Elemental mapping of $Na_{1.4}K_{0.6}Ni_2TeO_6$.

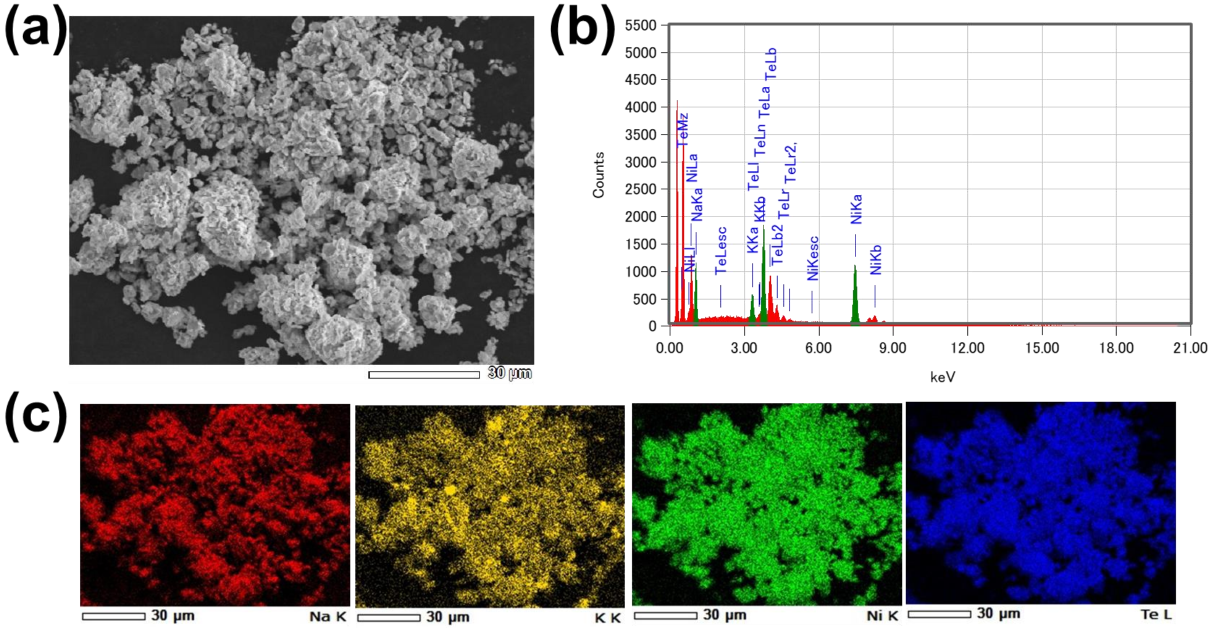

Supplementary Figure 7. (a) SEM picture of the $Na_{1.8}K_{0.2}Ni_2TeO_6$ powders. **(b)** SEM-EDX spectrum of the powders. **(c)** Elemental mapping of $Na_{1.8}K_{0.2}Ni_2TeO_6$.

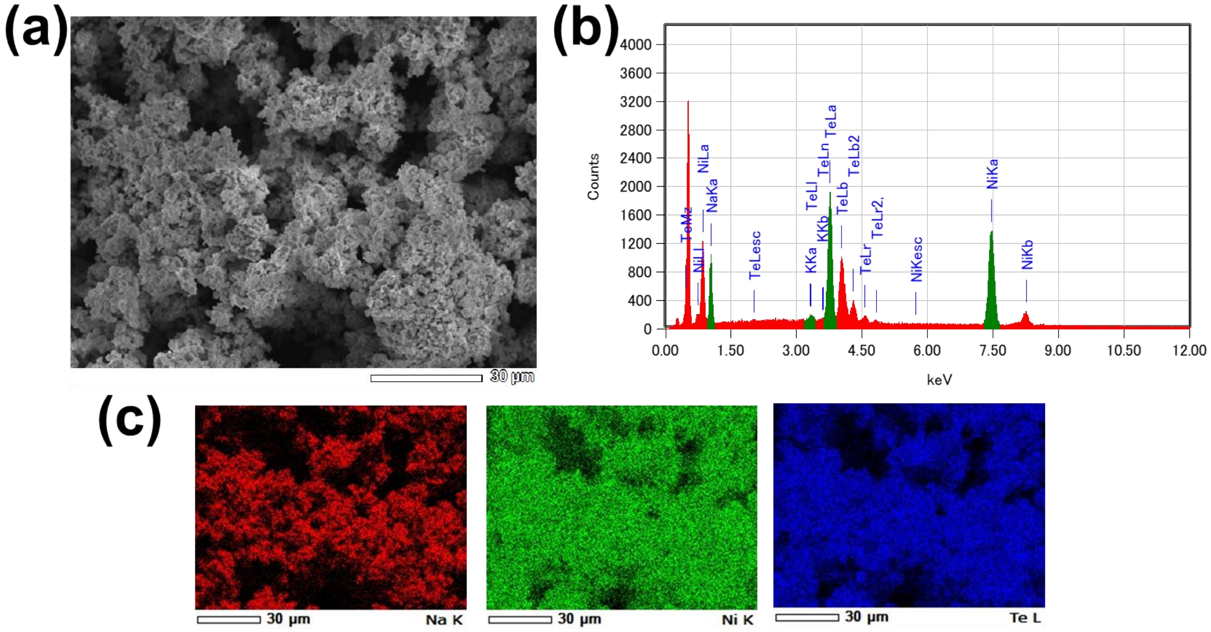

Supplementary Figure 8. (a) SEM picture of the $Na_2Ni_2TeO_6$ powders. **(b)** SEM-EDX spectrum of the powders. **(c)** Elemental mapping of $Na_2Ni_2TeO_6$.

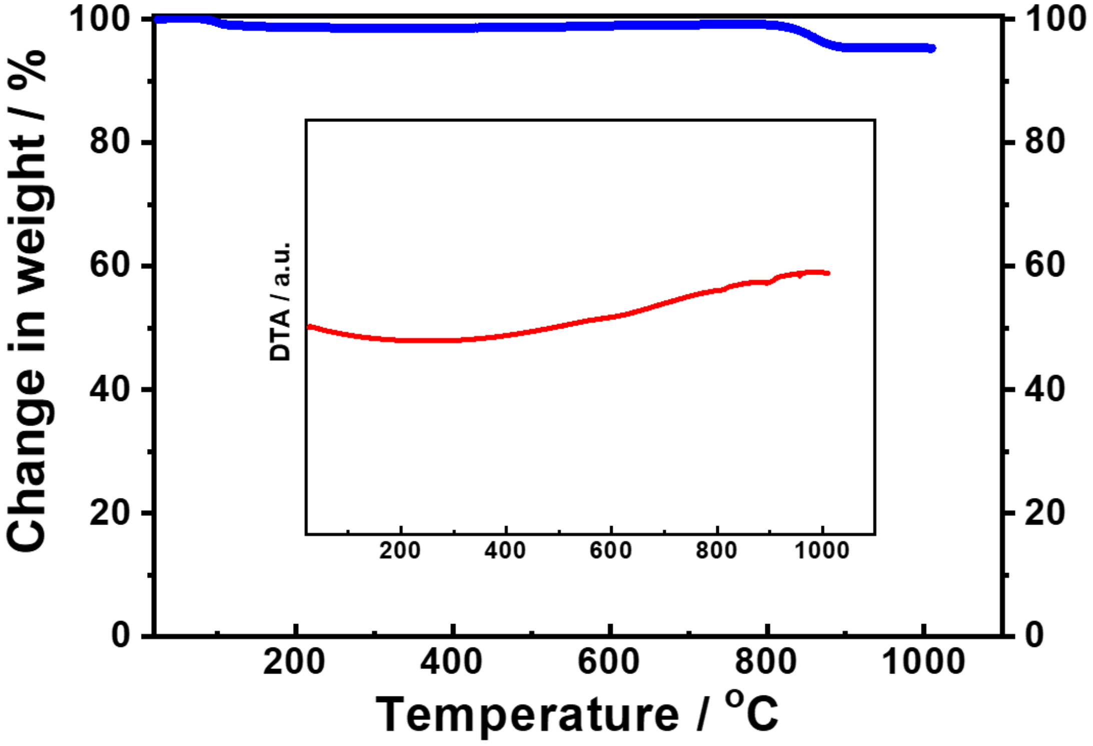

Supplementary Figure 9. Thermal gravimetric analyses curves of $NaKNi_2TeO_6$, showing its thermal stability to up to 800°C.

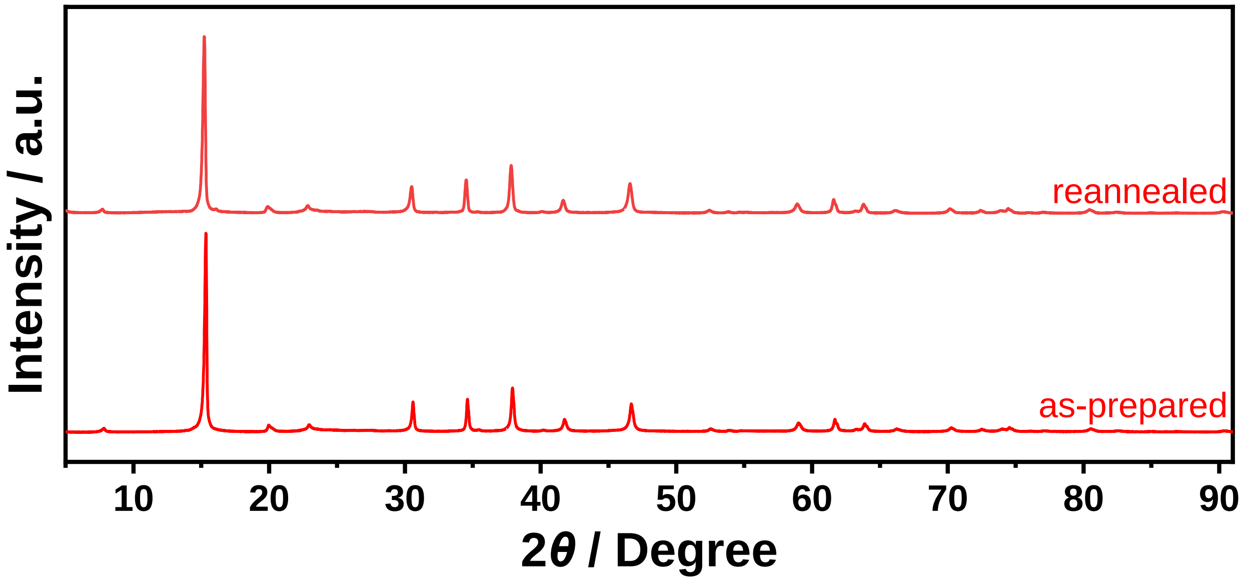

Supplementary Figure 10. A comparison of the XRD patterns of as-prepared and reannealed $NaKNi_2TeO_6$, revealing structural stability of $NaKNi_2TeO_6$ upon thermal treatment. The wavelength was set at Cu *K*α.

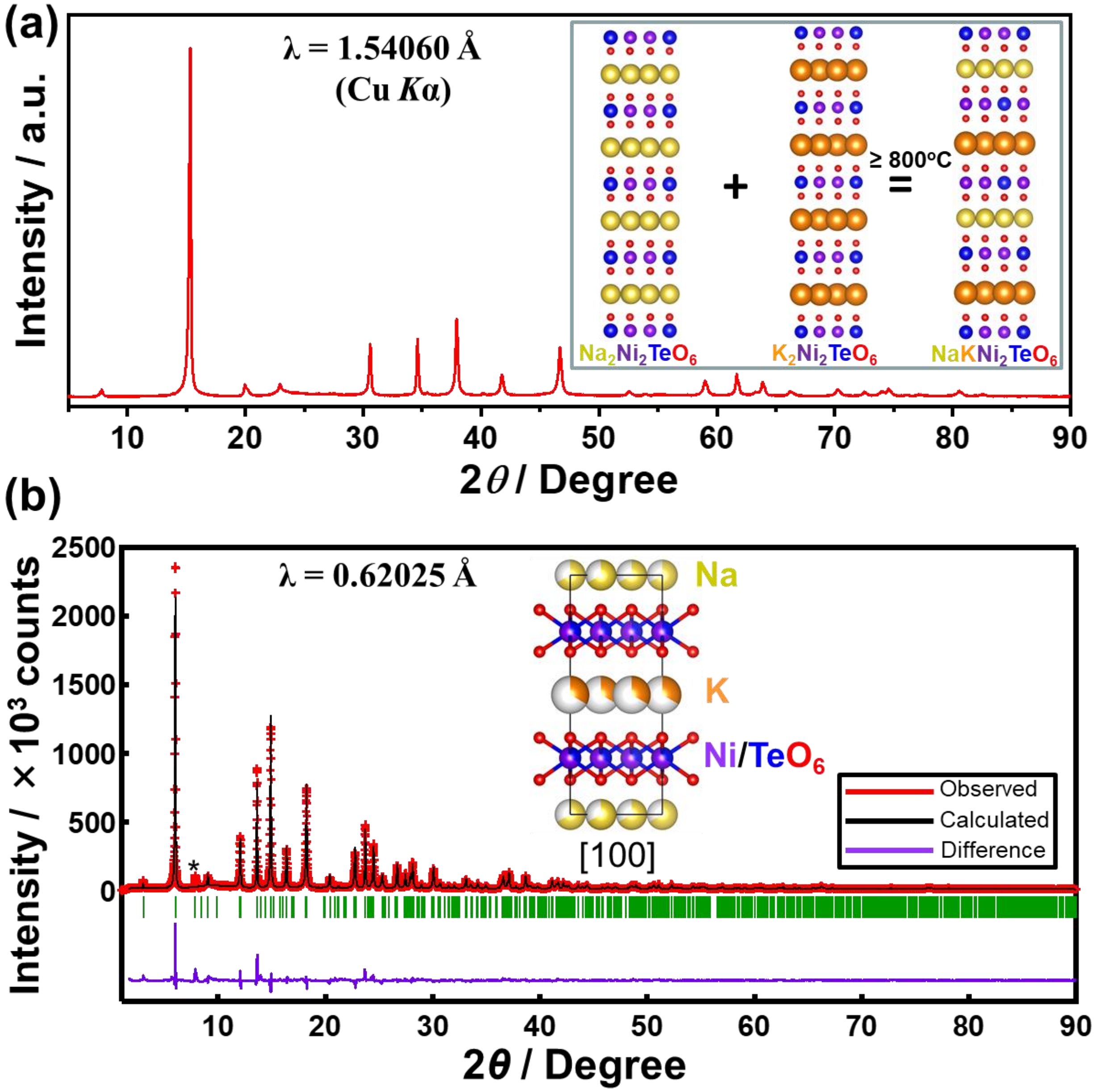

Supplementary Figure 11. Conventional XRD pattern of $NaKNi_2TeO_6$ prepared via high-temperature solid-state reaction of $Na_2Ni_2TeO_6$ and $K_2Ni_2TeO_6$. **(a)** Conventional XRD patterns reveal a highly crystalline sample, although some diffraction peaks are highly asymmetric and broad. Schematic showing the synthesis protocol is shown (in inset). **(b)** Rietveld refinement plots of the synchrotron XRD pattern of $NaKNi_2TeO_6$ indexed in a hexagonal cell ($P\overline{6}2m$ space group model (shown in inset)). Although most diffraction peaks are indexed using this model, a Bragg peak centered at 7.86^0 (shown in asterisk) is not fitted and the pattern appears not well matched. The refined lattice parameters are: a = 5.226(1) Å and c = 11.7840(4) Å. The isotropic atomic displacement parameters (U_{iso}) of the K1, K2, K3 sites were constrained. The z coordinates and U_{iso} of Ni1, Te1, Ni2, Te2 were also constrained. The reliability factors attained were as follows: R_{wp} = 11.56%, R_p = 8.22% and goodness-of-fit (GOF) = 5.31, warranting more analyses based on other structural models.

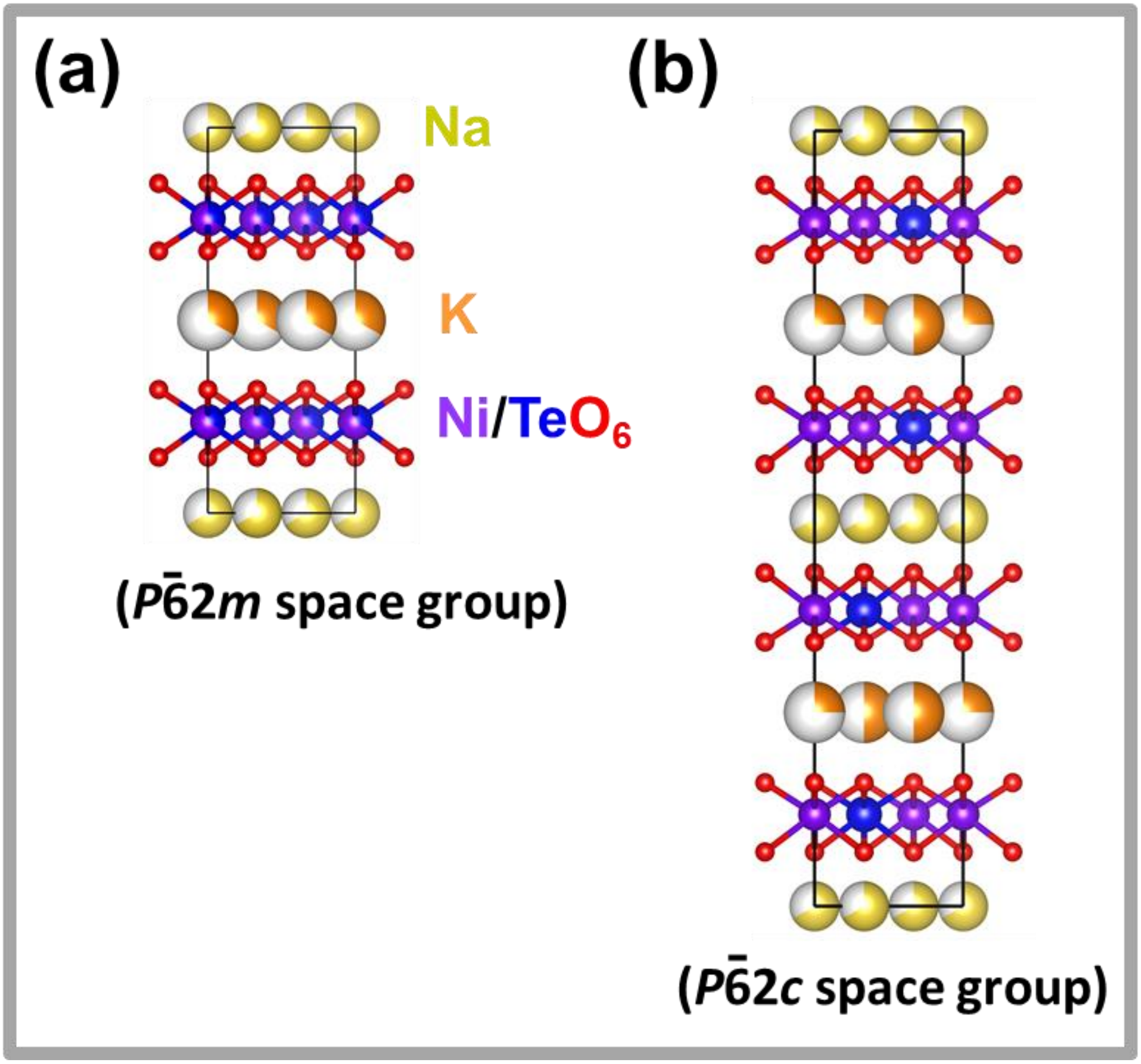

Supplementary Figure 12 Structural models (hexagonal space groups) used to index the XRD pattern of $NaKNi_2TeO_6$. **(a)** A hexagonal cell ($P\bar{6}2m$) model used to initially index the synchrotron XRD data of $NaKNi_2TeO_6$ shown in **Supplementary Figure 11**. **(b)** A model with double periodicity (indexed in $P\bar{6}2c$ space group).

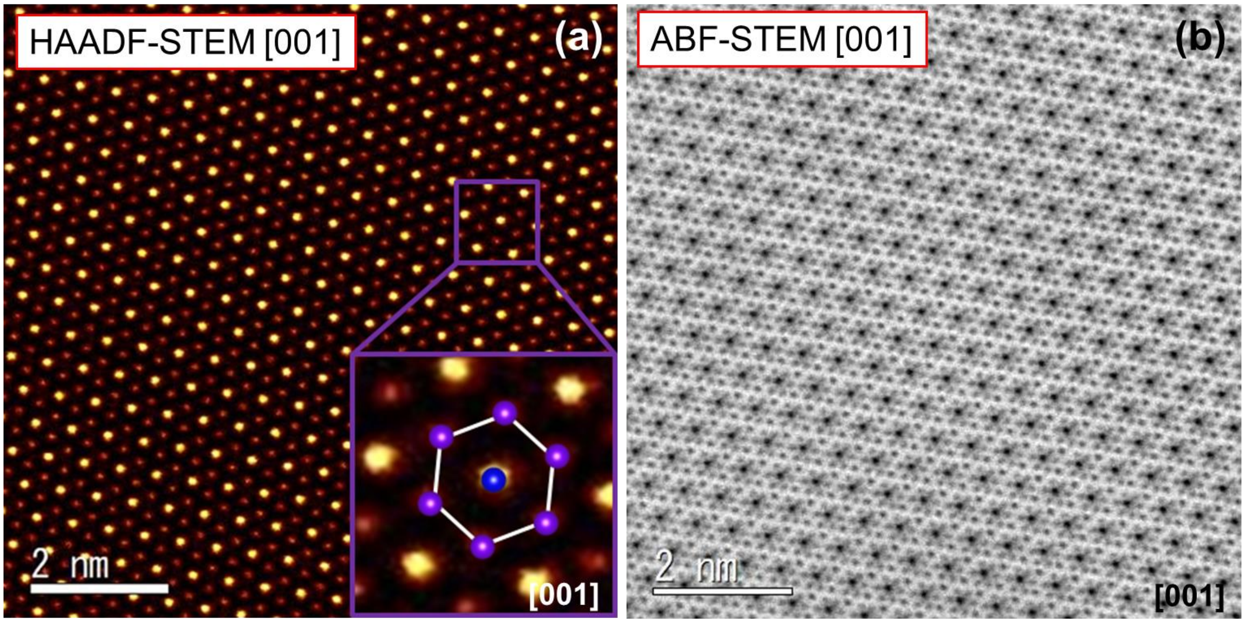

Supplementary Figure 13. (a) High-angle annular dark-field (HAADF) and **(b)** Annular bright-field (ABF) scanning transmission electron microscopy (STEM) images of $K_2Ni_2TeO_6$ taken along the [001] zone axis. Red spots mark columns of Ni atoms whilst bright yellow spots correspond to Te atoms columns. Distinct from the results illustrated by $NaKNi_2TeO_6$ in **Figure 2** of the manuscript, the honeycomb configuration of Ni atoms around Te can be discerned readily.

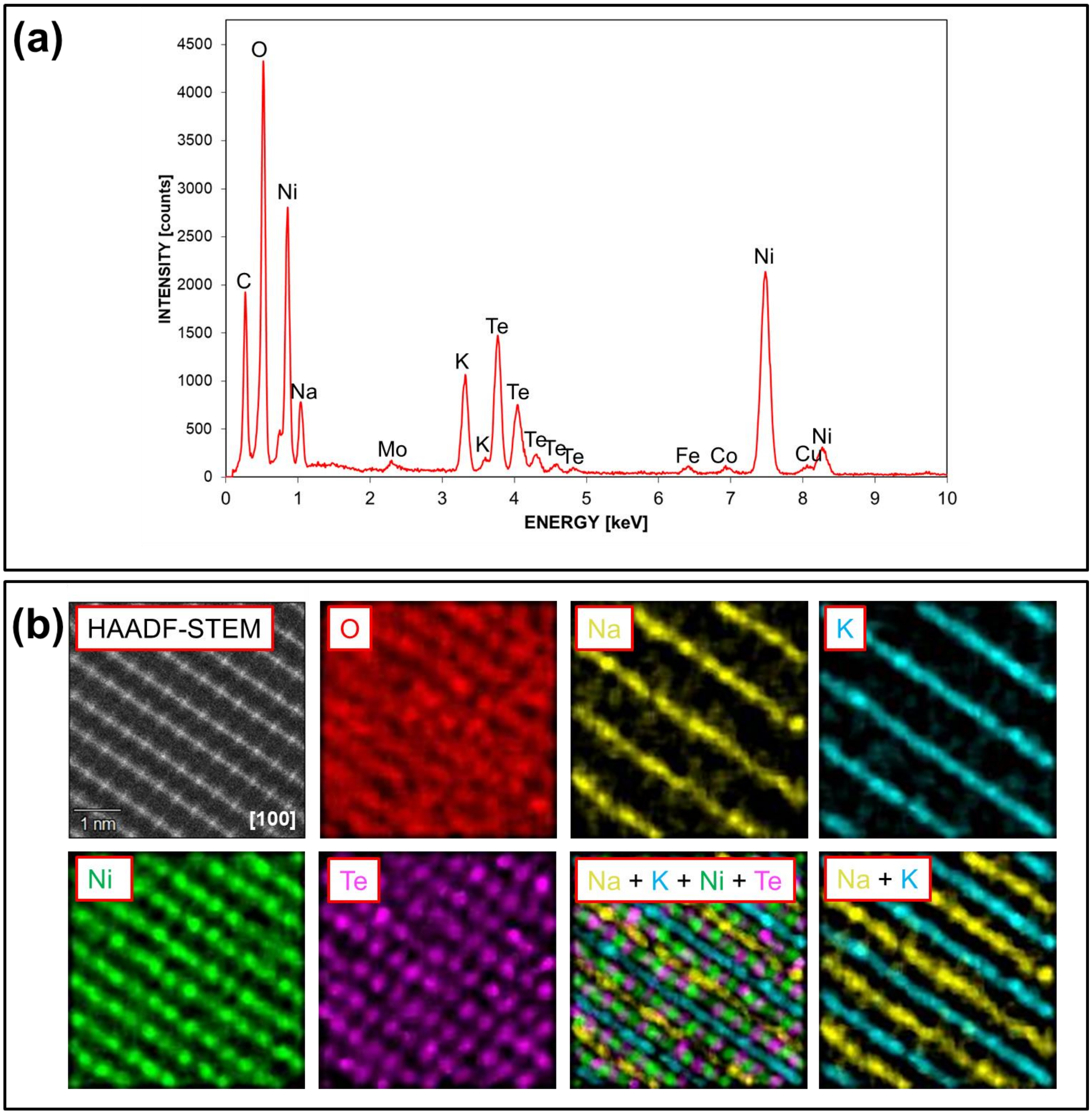

Supplementary Figure 14. Elemental analysis of $NaKNi_2TeO_6$ using STEM-energy dispersive X-ray (EDX) spectroscopy. **(a)** EDX spectrum, where energies corresponding to the relevant elements are marked. C, Mo, Fe and Cu peaks emanate from the sample holder. **(b)** HAADF-STEM images taken along the [100] zone axis and the corresponding elemental mapping acquired from the same area, where different colours highlight the distribution of the elements.

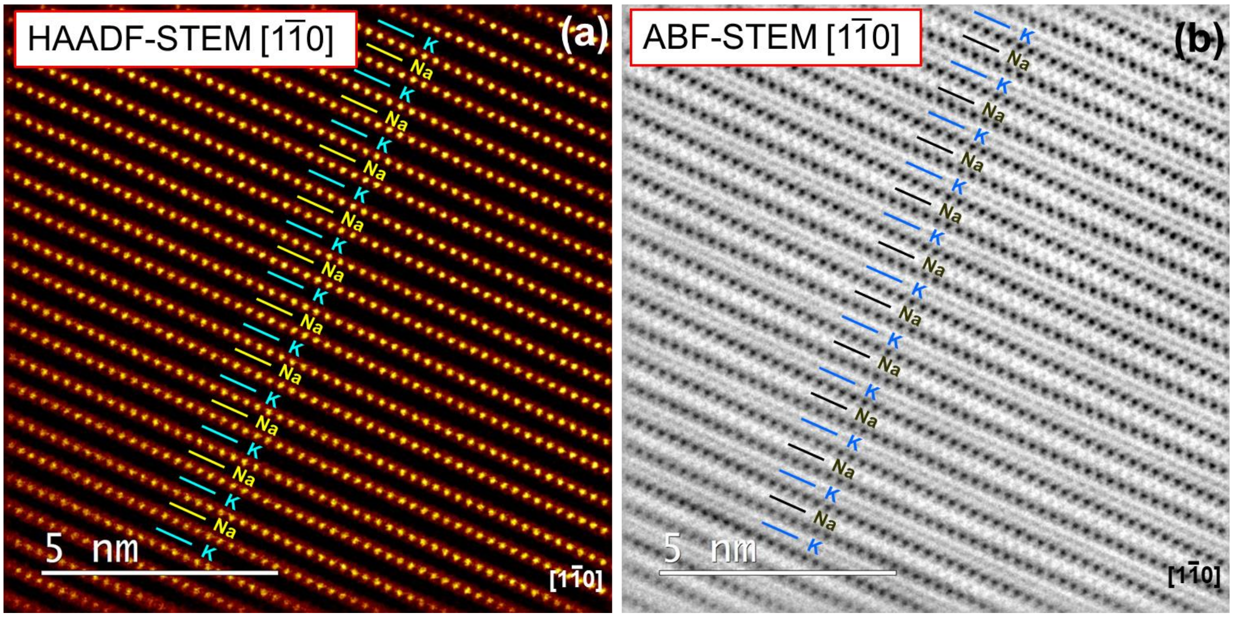

Supplementary Figure 15. STEM images of $NaKNi_2TeO_6$ taken along the [1$\bar{1}$0] direction. **(a)** High-angle annular dark-field (HAADF) STEM images showing the alternating arrangement of K and Na layers. The bright spots in the HAADF-STEM image correspond to Te and Ni atoms residing in the honeycomb slabs. **(b)** Corresponding ABF-STEM images where the Te and Ni atoms are indicated by the dark spots. The Na, and K atoms can also be observed as light grey sections located between Te/Ni slabs. The arrangement of K and Na atoms is distinct, indicating that the crystallographic sites occupied by Na differ from those occupied by K. Moreover, the intensity distribution is different even within the layers comprising Na and K, indicating variation in the occupancy of Na and K in their respective sites.

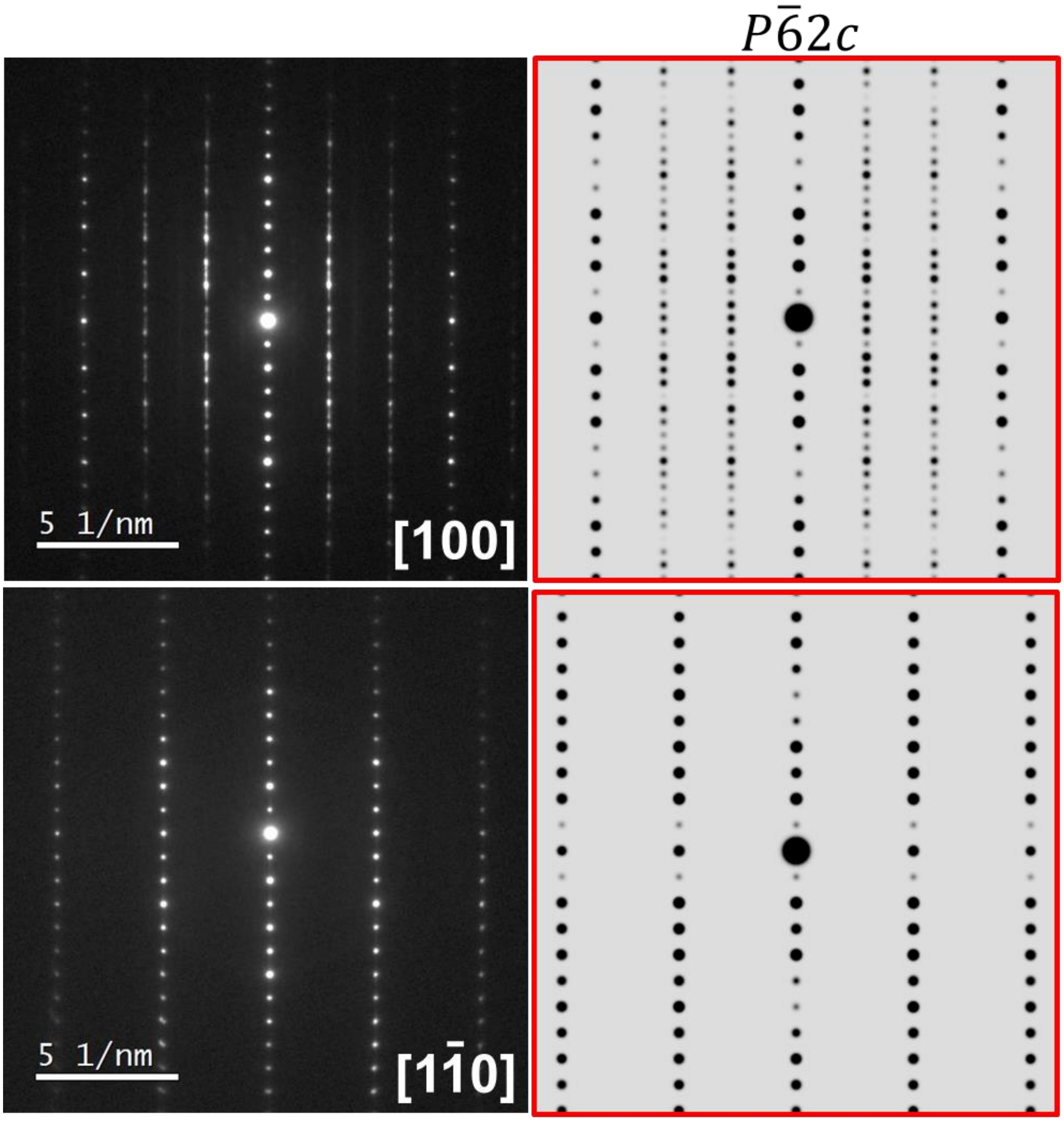

Supplementary Figure 16. Comparison of the selected area electron diffraction (SAED) patterns of $NaKNi_2TeO_6$ experimentally taken with those of the proposed structural models, indicating the $P\bar{6}2c$ double periodicity hexagonal model as the most appropriate to describe the structure of $NaKNi_2TeO_6$.

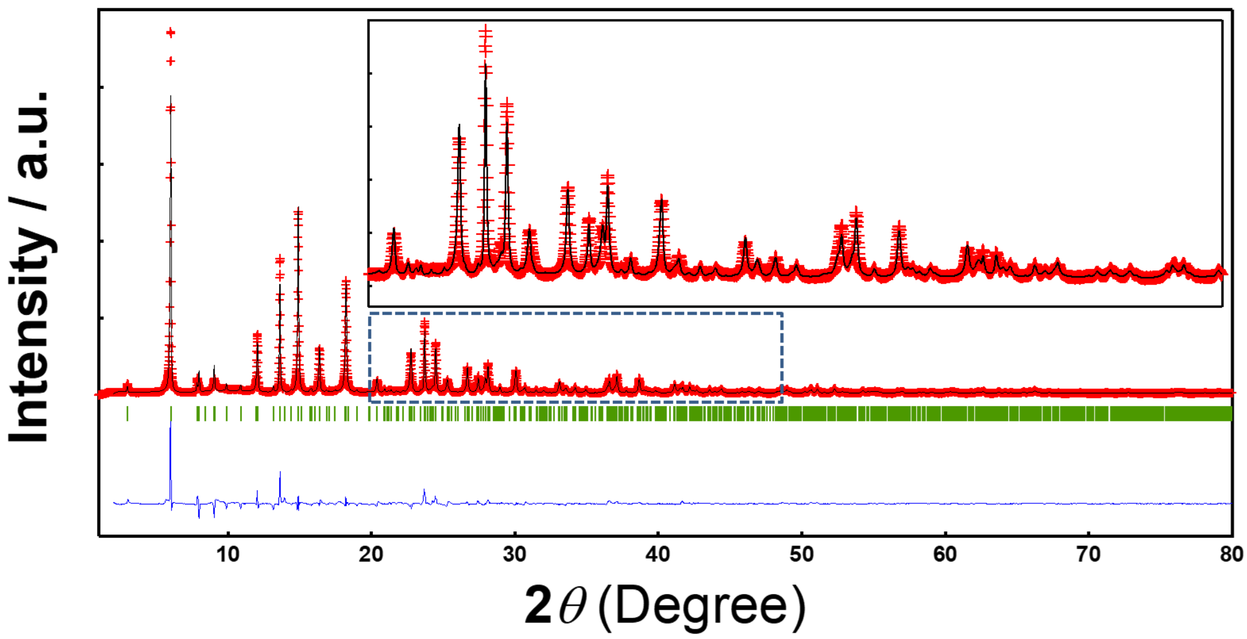

Supplementary Figure 17. Rietveld refinement plots of the synchrotron XRD pattern of $NaKNi_2TeO_6$ indexed in a hexagonal cell ($P\overline{6}2c$ space group model (shown in **Supplementary Figure 12**)). The reliability factors attained were as follows: R_{wp} = 13.84%, R_p = 9.57% and GOF = 6.37, warranting more analyses based on transmission electron microscopy. The wavelength was set at 0.62025 Å.

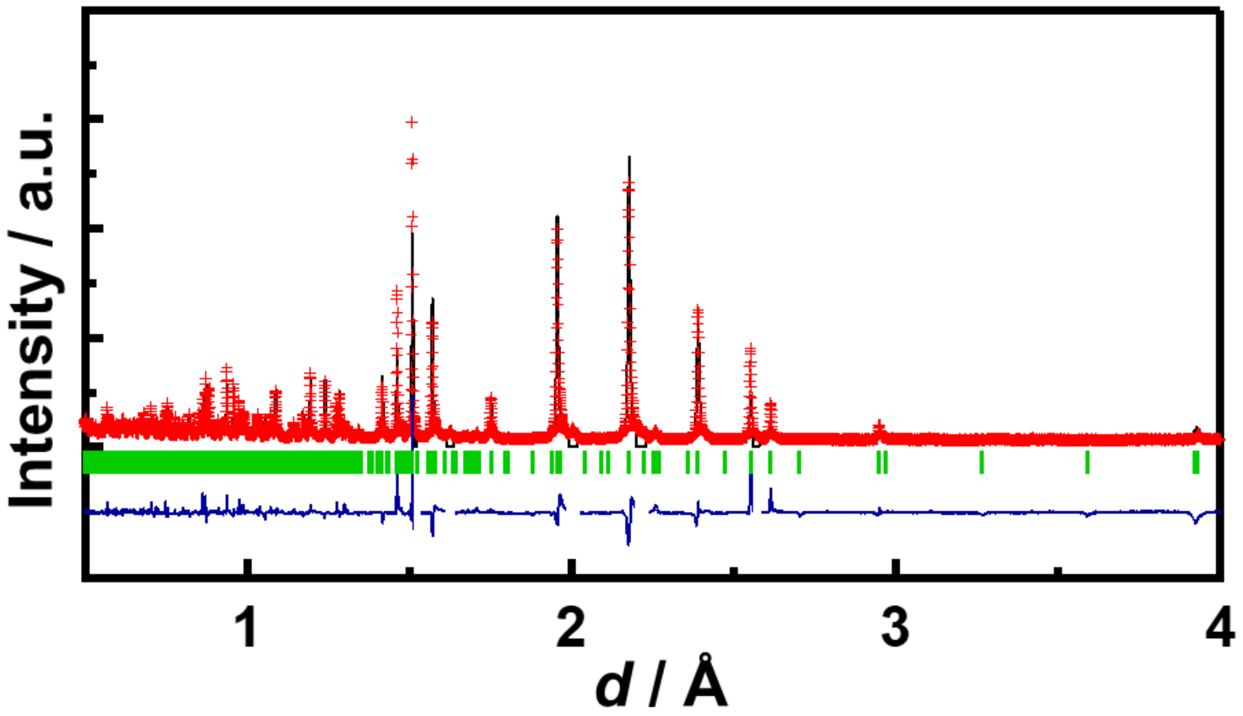

Supplementary Figure 18. Rietveld refinement plots of the neutron diffraction (ND) pattern of $NaKNi_2TeO_6$ indexed in a hexagonal cell ($P\overline{6}2c$ space group model (shown in **Supplementary Figure 12**)). Peaks that could not be well-fitted in the following *d* ranges were excluded from the refinement: 1.513–1.522 Å, 1.614–1.636 Å, 1.990–2.016 Å, 2.200–2.228 Å, and 2.560–2.578 Å. Several issues with peak intensity and asymmetric peak profile as noted in the SXRD data, which arise from the stacking disorder of the [Ni_2Te] layers, are apparent also in the ND refinement. The reliability factors attained were as follows: R_{wp} = 10.76%, R_p = 8.92%, GOF = 6.78.

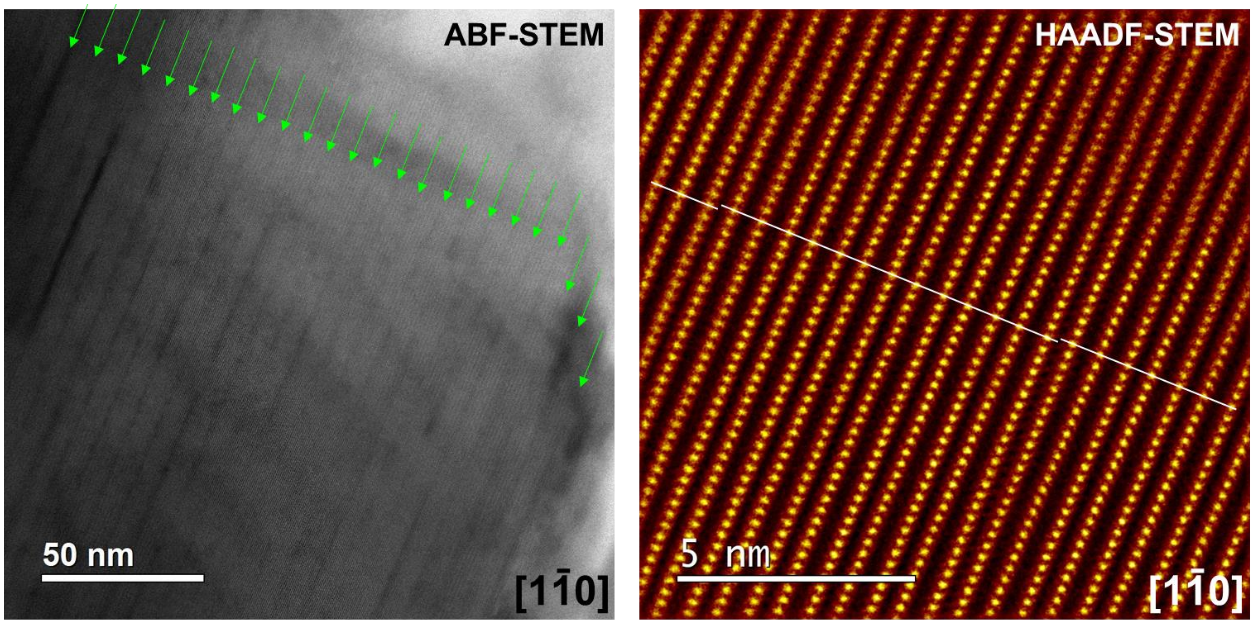

Supplementary Figure 19. Direct visualisation of stacking defects along the *c*-axis [001] direction. **(left)** Low magnification ABF-STEM images showing streaks indicative of planar defects in $NaKNi_2TeO_6$. **(right)** High magnification HAADF-STEM images showing shifts in the Te/Ni slab at regions where the stacking faults are found to occur in $NaKNi_2TeO_6$. FAULTS program was further employed to quantitatively assess the nature of these stacking faults, results of which are shown in **Supplementary Figure 20**.

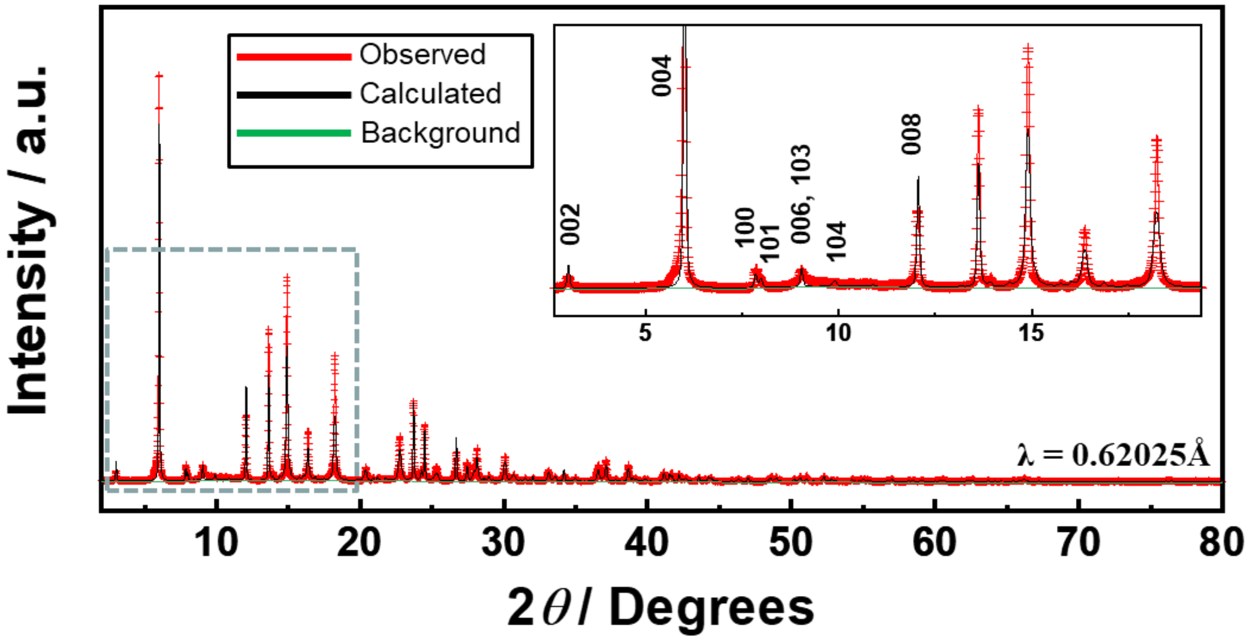

Supplementary Figure 20. Refined synchrotron XRD pattern of $NaKNi_2TeO_6$, used to perform quantitative analyses of stacking faults with the FAULTS program. An enlarged image of the low-angle Bragg diffraction peaks is highlighted. The final lattice parameters obtained are as follows: a = 5.2269(2) Å and c = 23.5962(2) Å with a goodness-of-fit (GOF) value of 1.604. Stacking vectors having a magnitude of [−1/3, −1/3, 0], [1/3, 0, 0] and [0, 1/3, 0] were found to be dominant with stacking probabilities of 7.5%, 6.3% and 4.1%, respectively.

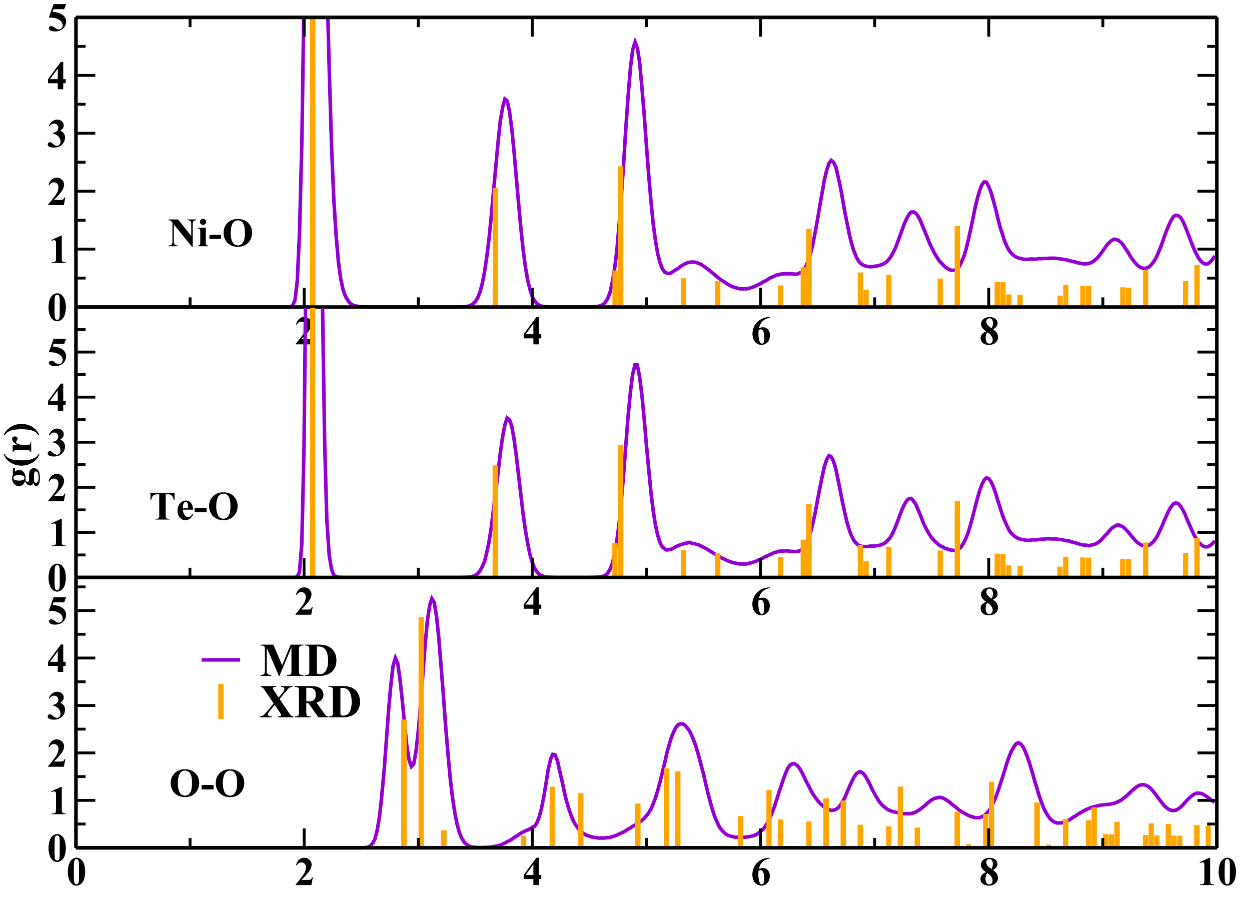

Supplementary Figure 21. Radial distribution function, g(r), between selected ion pairs (Ni−O, Te−O and O−O) for $NaKNi_2TeO_6$ at 600 K, calculated from isothermal-isobaric ensemble molecular dynamics (NPT-MD). The vertical bars (in orange) are the corresponding radial distribution functions (g(r)) based on the room-temperature X-ray structure. Note that the intensity has been rescaled for clarity.

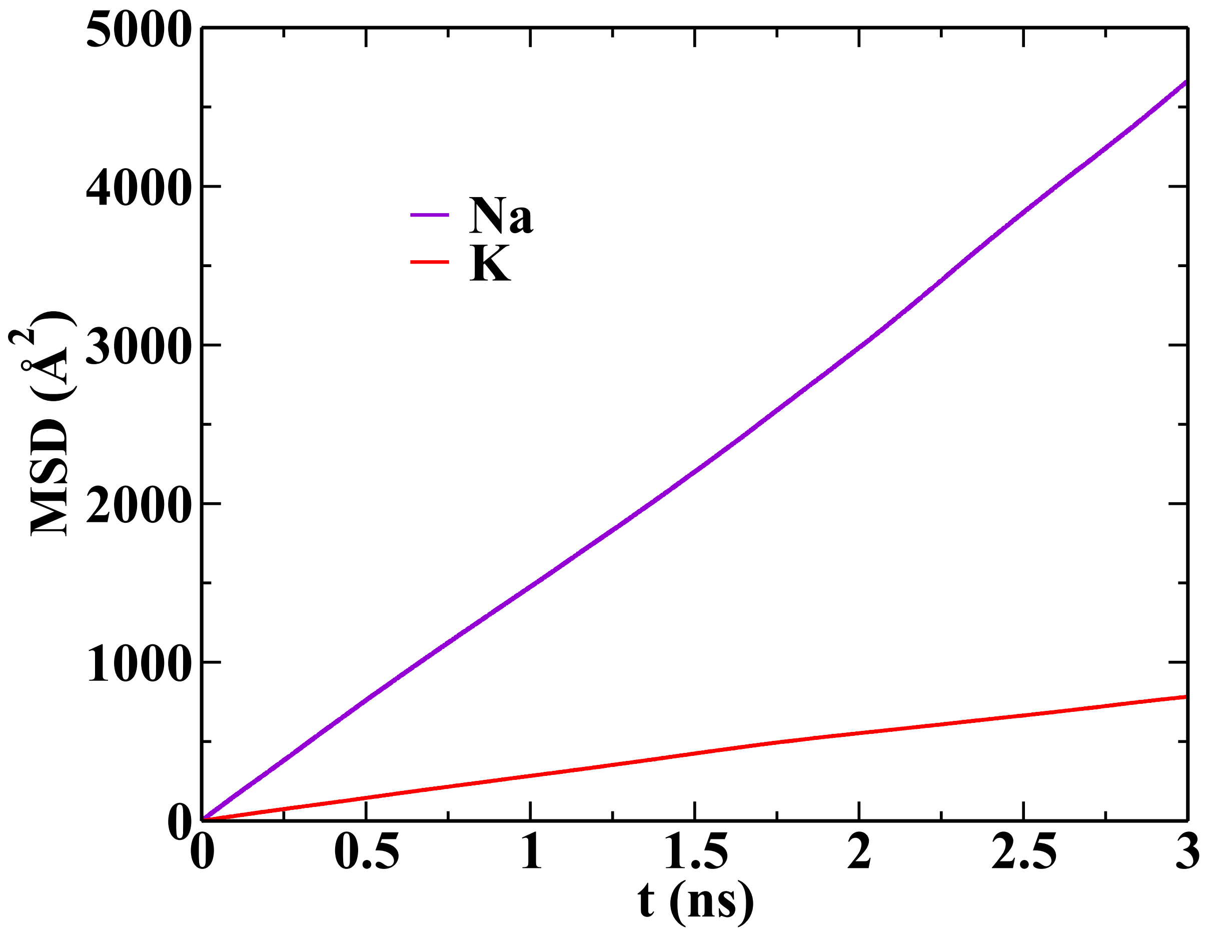

Supplementary Figure 22. Mean square displacement (MSD) of Na^+ and K^+ ions as a function of time (t) in $NaKNi_2TeO_6$, simulated at 600 K based on molecular dynamics (MD).

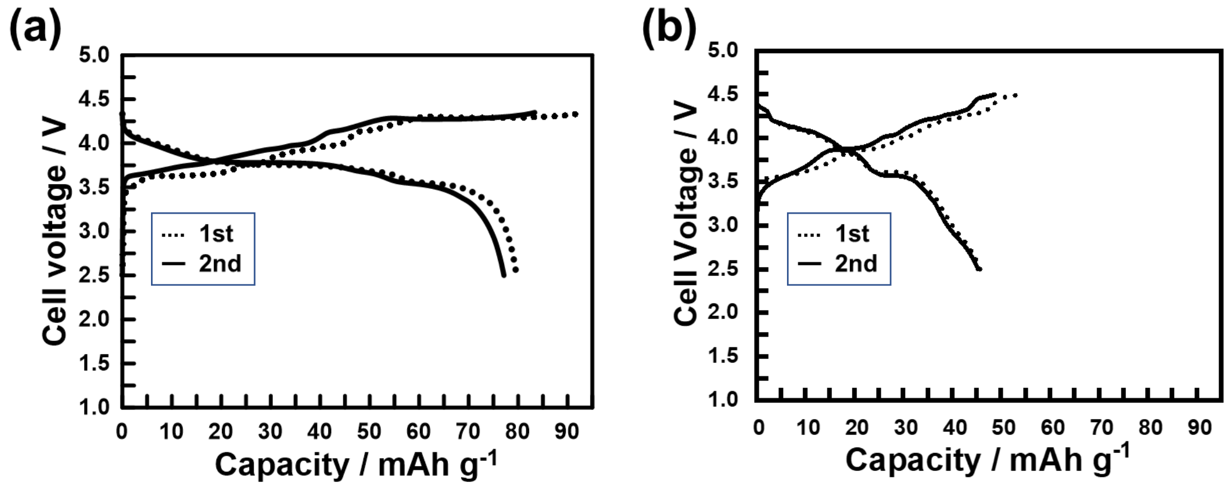

Supplementary Figure 23. Voltage–capacity profiles of $NaKNi_2TeO_6$ in **(a)** Na half-cells and **(b)** K half-cells performed in a lower cut-off voltage of 2.5 V at room temperature and under a specific current of 6.65 mA g^{-1}. The capacity can still be maintained by adjusting the lower cut-off voltage to 2.5 V. The first and second voltage profiles are indicated in dashed and continuous lines, respectively.

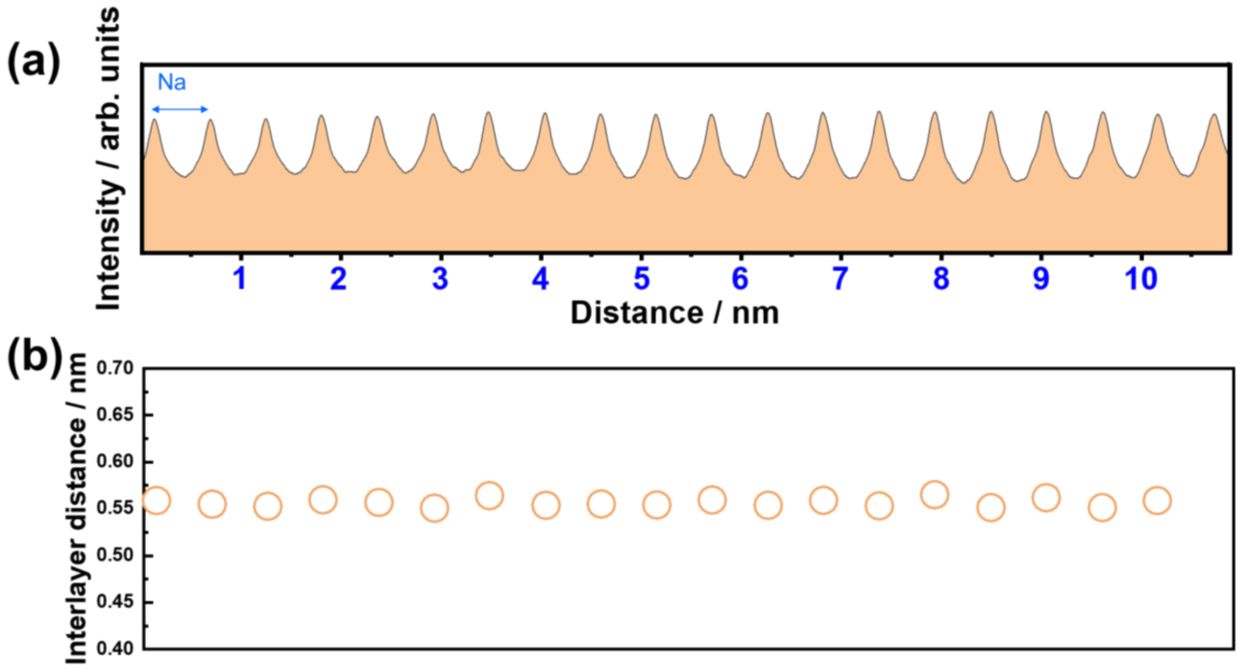

Supplementary Figure 24. Structural changes during alkali-ion (de)insertion of $NaKNi_2TeO_6$ upon subsequent cycling in Na half-cells. **(a)** Intensity line profiles showing equidistant interlayer spacings (0.55 nm) of Na atoms along the [001] axis as quantitatively illustrated in **(b)**. For clarity, the horizontal axis in (b) shows the layer numbers.

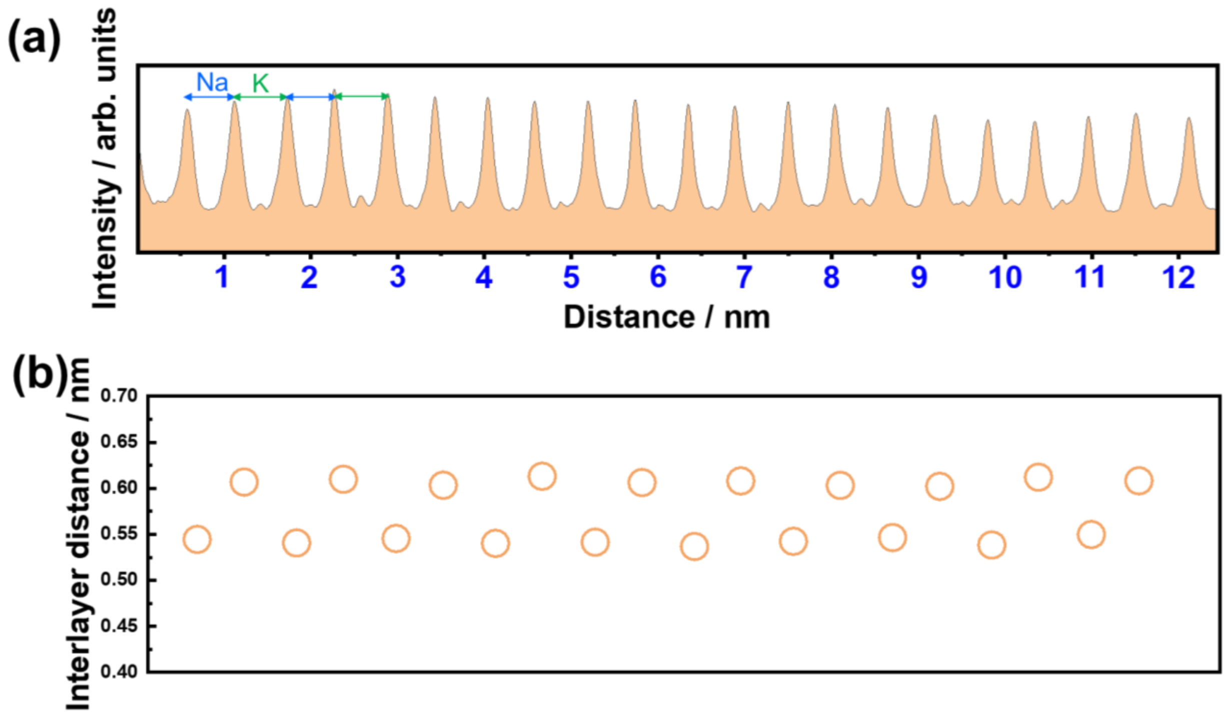

Supplementary Figure 25. Structural changes during alkali-ion (de)insertion of $NaKNi_2TeO_6$ upon subsequent cycling in K half-cells. **(a)** Intensity line profiles showing alternating interlayer spacings (0.55 nm and 0.62 nm) of Na and K atoms, respectively, along the [001] axis. This is further quantitatively illustrated in **(b)**. For clarity, the horizontal axis in **(b)** shows the layer numbers.

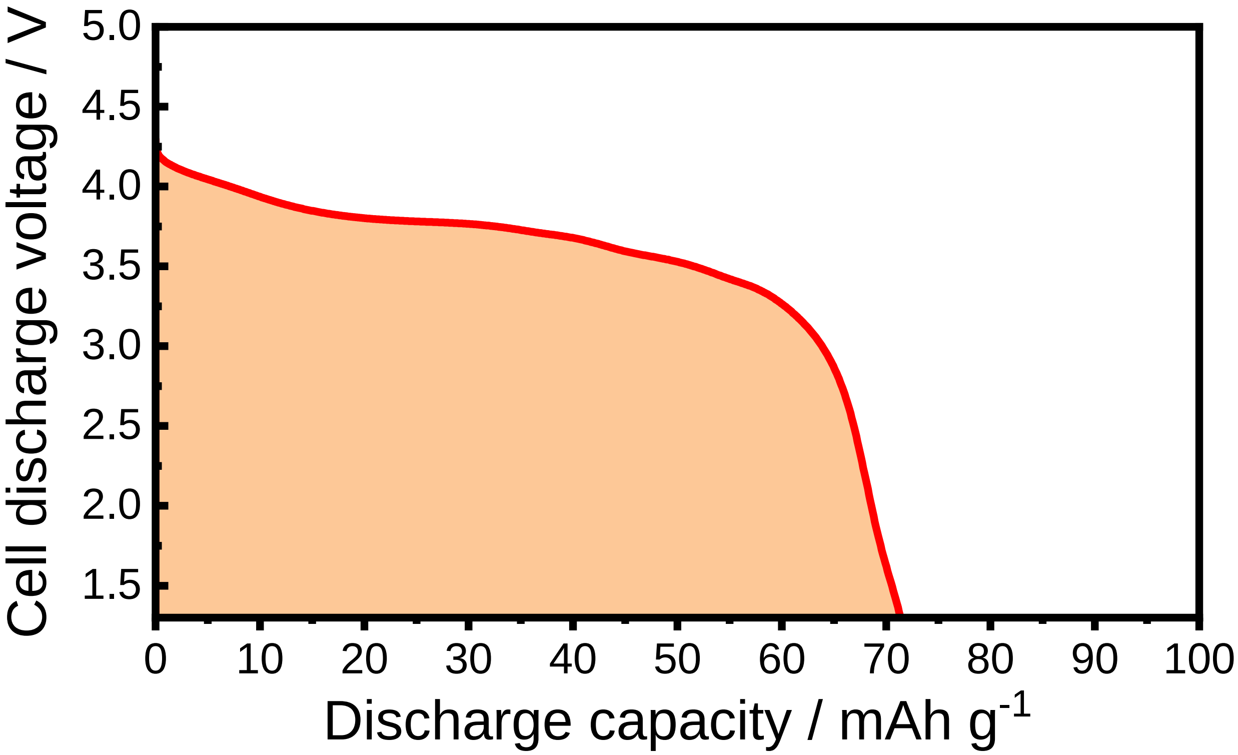

Supplementary Figure 26. Voltage-capacity profile of $NaKNi_2TeO_6$ (in NaK cell) initially discharged at a specific current of 13.4 mA g^{-1}. The average discharge voltage was calculated by dividing the area of the discharge curve (highlighted in ochre in the voltage-capacity plot) by the capacity value obtained when the cell reached the lower cut-off voltage. The average discharge voltage was calculated to be 3.752 V.

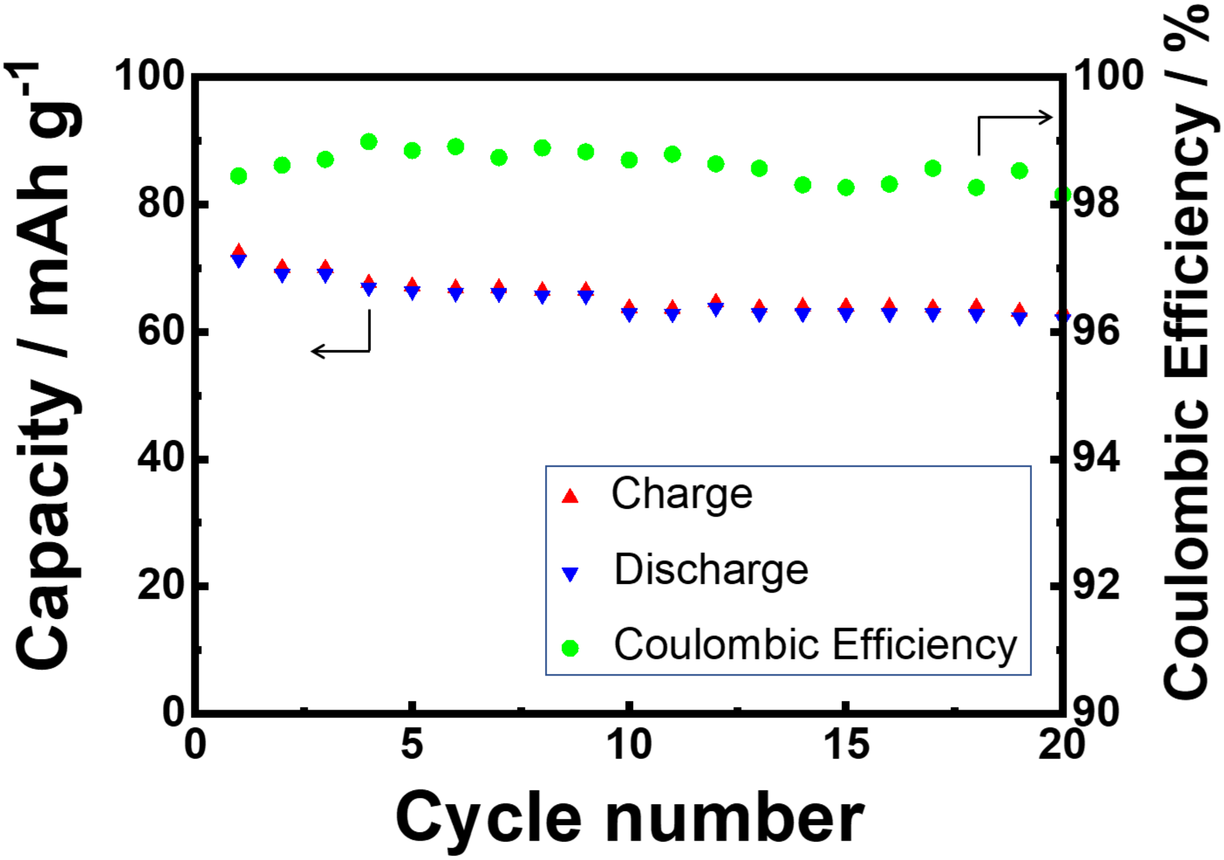

Supplementary Figure 27. Capacity and coulombic efficiency plotted as a function of the number of cycles for $NaKNi_2TeO_6$ in NaK cell. Coulombic efficiency was calculated as the ratio between discharge and charge capacities multiplied by 100%. Galvanostatic measurements were performed at a specific current of 13.4 mA g^{-1}.

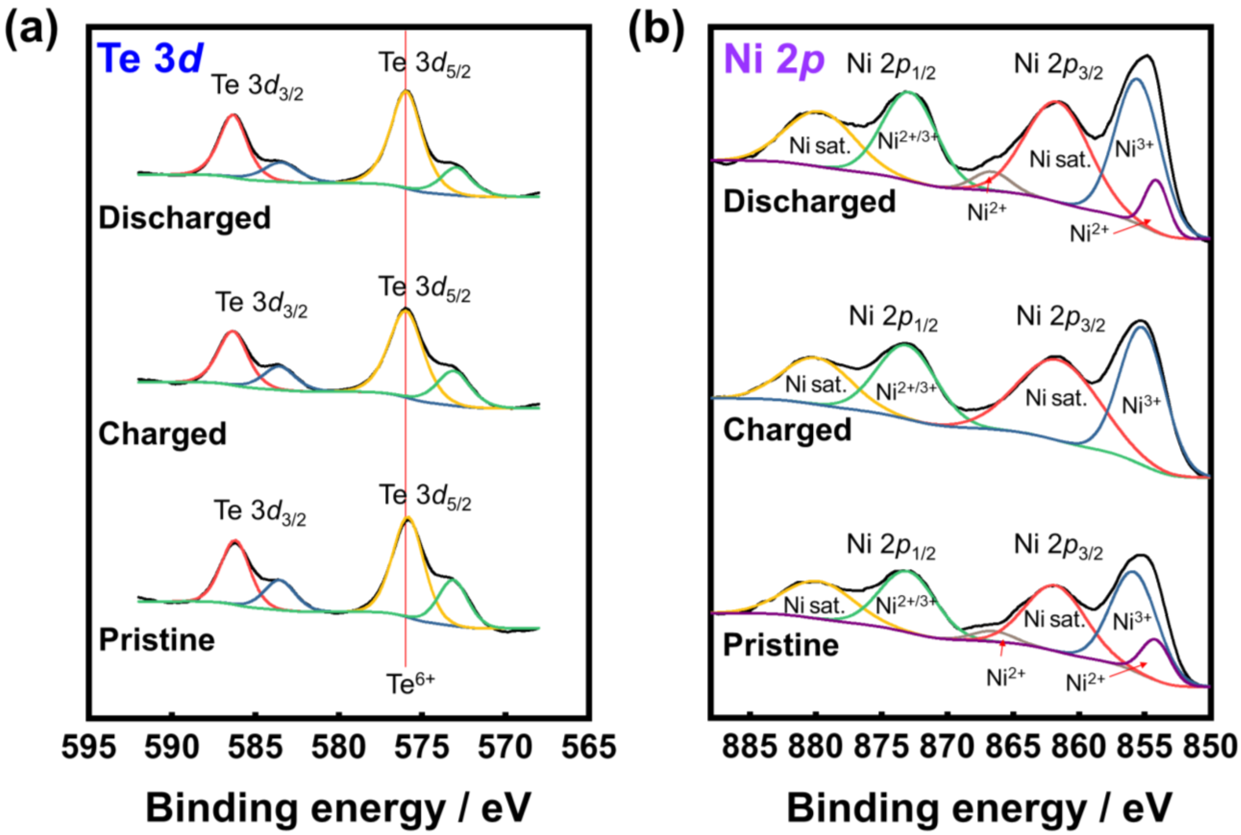

Supplementary Figure 28. Electronic structural changes during alkali-ion (de)insertion of $NaKNi_2TeO_6$ upon subsequent cycling in NaK half-cells. **(a)** Te 3*d* and **(b)** Ni 2*p* XPS spectra taken for the pristine, charged and discharged electrodes. The satellite peaks are marked as 'Ni sat.'.

Looking at the Te 3*d* core spectra (**Supplementary Figure 28a**), the Te $3d_{5/2}$ peak centered at around 576.0 eV (which is characteristic of Te^{6+}) remains invariant for the entire charge–discharge process. This indicates that Te, as a spectator ion, does not participate in the redox process. This has also been observed upon alkali-ion extraction and reinsertion in related honeycomb layered oxides such as $K_2Ni_2TeO_6$ upon charging and discharging. Turning to the Ni 2*p* core spectra (**Supplementary Figure 28b**), *vide infra*, changes are discernible indicative of the participation of the Ni 2*p* orbitals in the charge compensation process. Although the Ni^{3+} at the electrode surface obfuscates the complete capture of the Ni^{2+} peak signal, a reversible oxidation of Ni^{2+} during charging and a reduction of Ni^{3+} during discharging is evident. Ni^{2+} is evinced by the main peak at 854.0 eV whereas the peak at 861.6 eV emanates from the auger spectra of Ni $2p_{3/2}$ (denoted as satellite peaks). The binding energy of the Ni $2p_{3/2}$ peak shifts to a higher value of 856.4 eV, which corresponds to Ni^{3+} after charging. Upon discharging, the Ni $2p_{3/2}$ peak signal can be gleaned, confirming that Ni 2*p* takes part in the charge compensation process. Although the bulk-sensitive XAS measurements are beyond the scope of the current work, the charge compensation process in $NaKNi_2TeO_6$ can be envisioned to be similar to that reported in related nickel tellurates such as $K_2Ni_2TeO_6$.

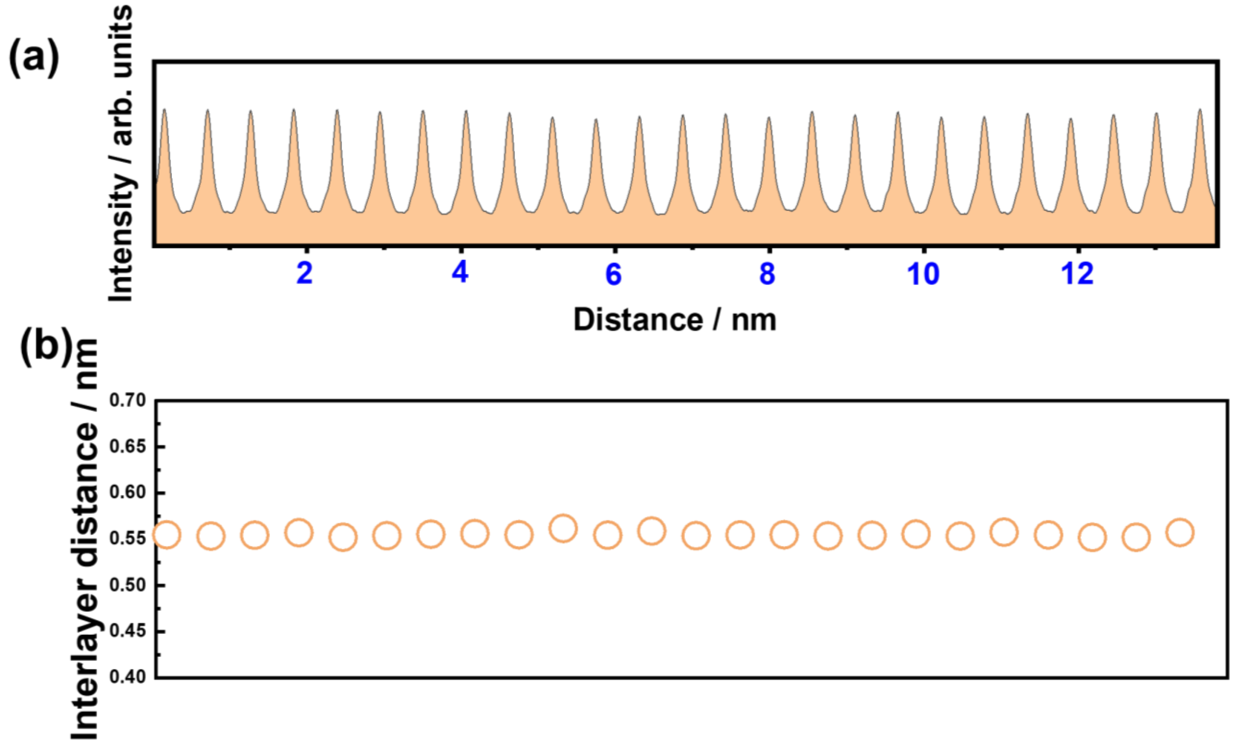

Supplementary Figure 29. Structural changes during alkali-ion (de)insertion of $NaKNi_2TeO_6$ upon subsequent cycling in NaK half-cells. **(a)** Intensity line profiles showing equidistant interlayer spacings (0.55 nm) along the [001] axis revealing $NaKNi_2TeO_6$ to preferentially insert Na atoms into the lattice. This is further quantitatively illustrated in **(b)**. For clarity, the horizontal axis in **(b)** shows the layer numbers.

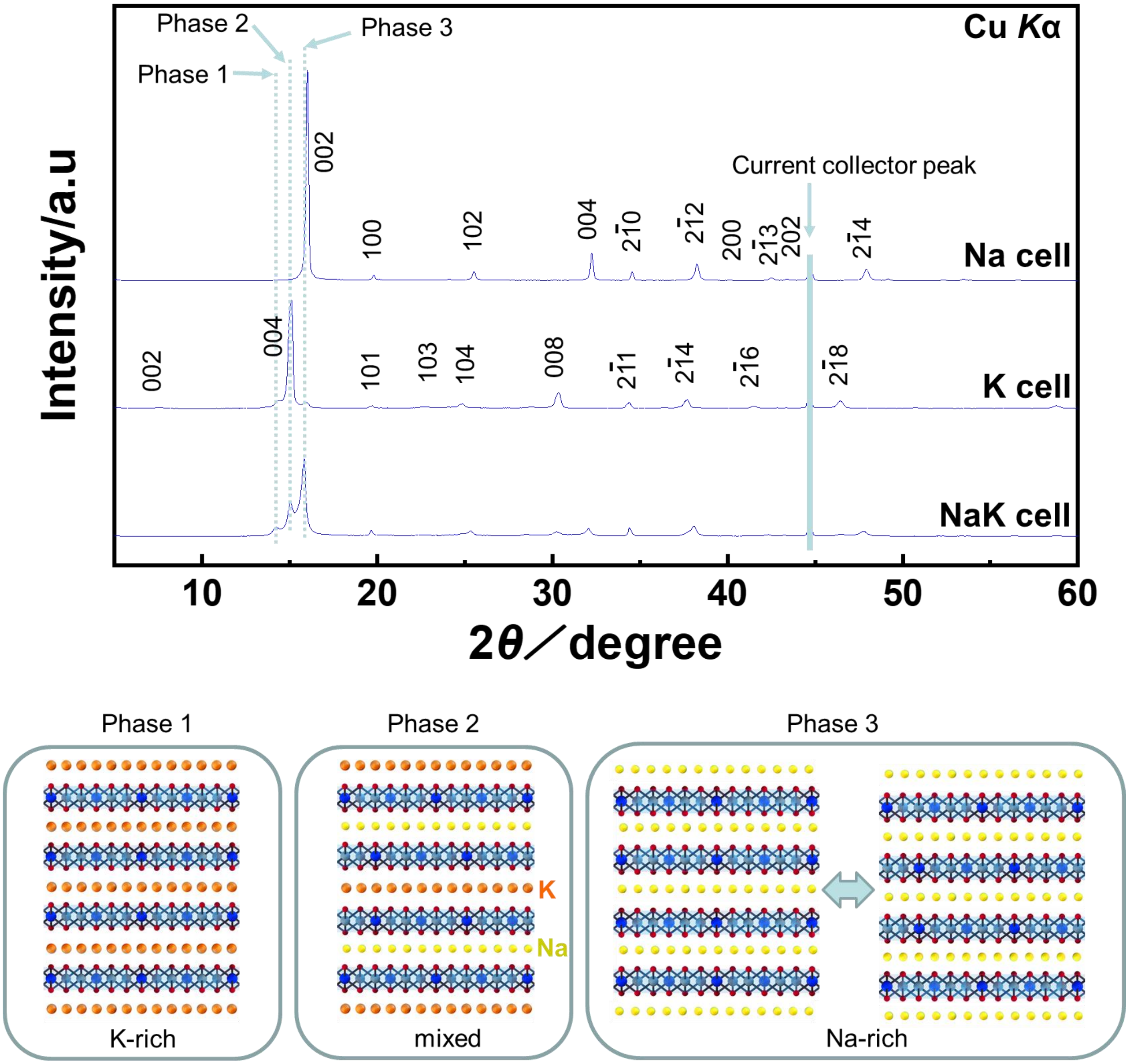

Supplementary Figure 30. Crystal structural changes during alkali-ion (de)insertion of $NaKNi_2TeO_6$ upon subsequent cycling in Na, K and NaK half-cells based on XRD measurements. In a NaK battery system, there are Na-rich domains, K-rich domains and mixed NaK domains. The proportion of Na-rich domains are largest, as revealed by the high intensity profiles of the XRD patterns of $Na_2Ni_2TeO_6$. The TEM micrographs for the Na-rich have been shown in **Figure 7** of the manuscript, as it manifests interesting defect structures worthy to highlight.

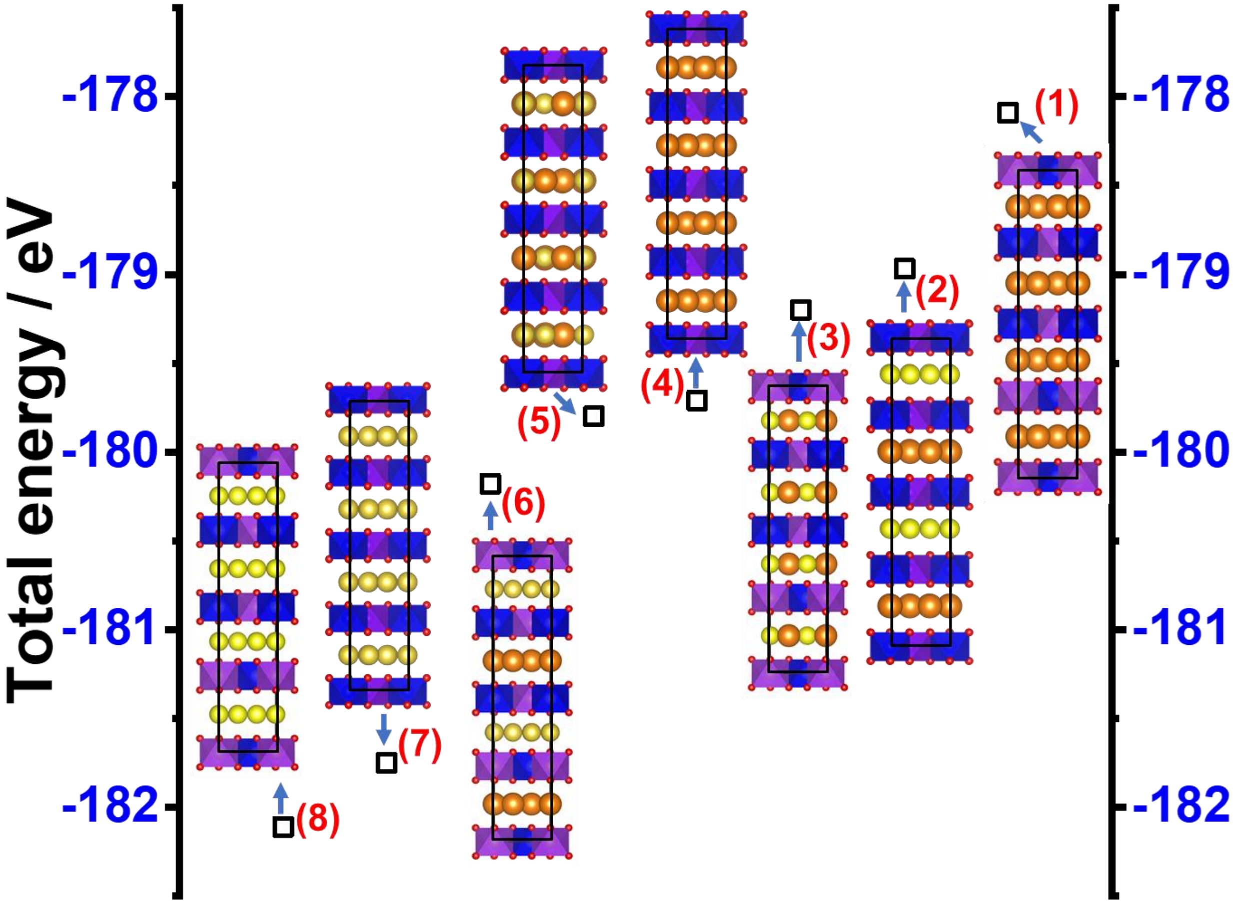

Supplementary Figure 31. Total formation energies for various configurations of $NaKNi_2TeO_6$ (2, 3, 5 and 6, shown in **Supplementary Figure 32**) and parent phases (1, 4, 7 and 8, shown in **Supplementary Figure 33**) based on DFT calculations. The experimentally attained configuration (structure 6) is the most stable amongst others calculated (for instance, structures 5, 3 and 2).

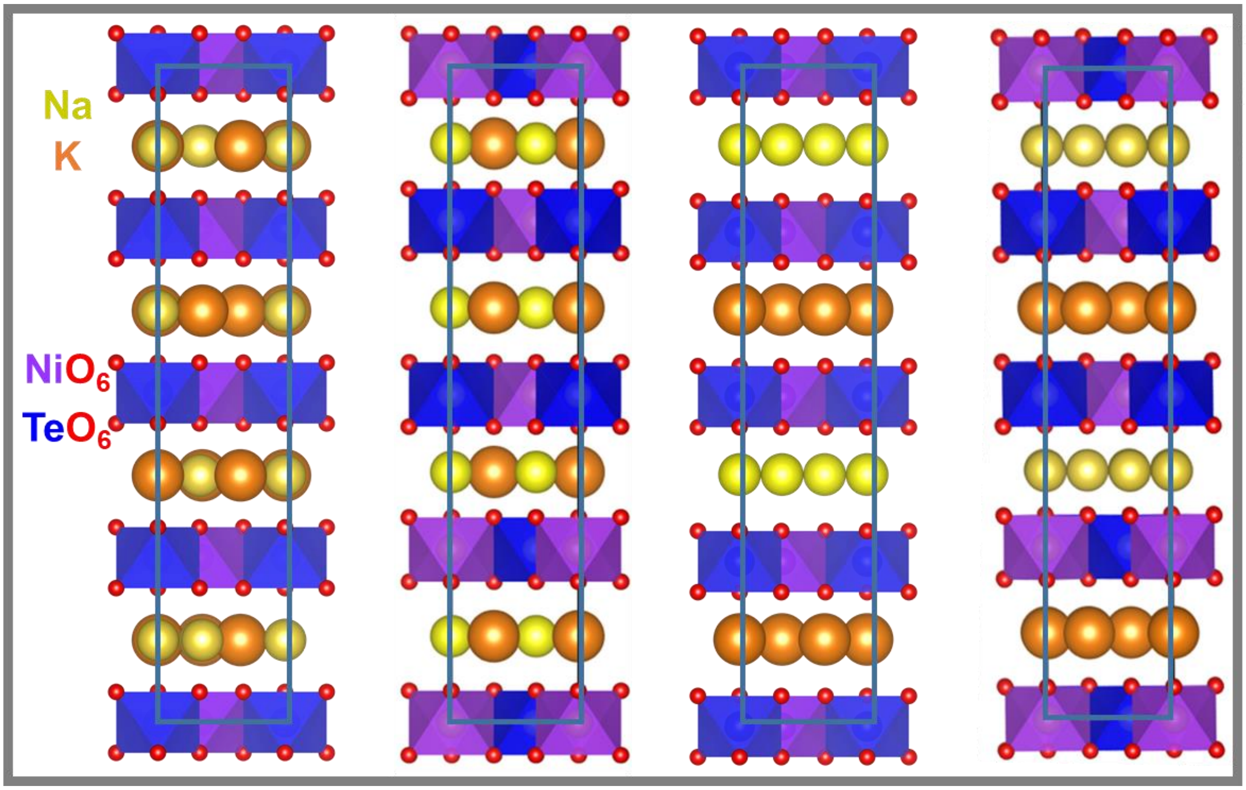

Supplementary Figure 32. Exemplar configurations of intermediate $NaKNi_2TeO_6$ compositions (with varying shifts of the Te/Ni slabs where Na atoms reside) assessed using theoretical computation. Their formation energies were calculated based on DFT calculations.

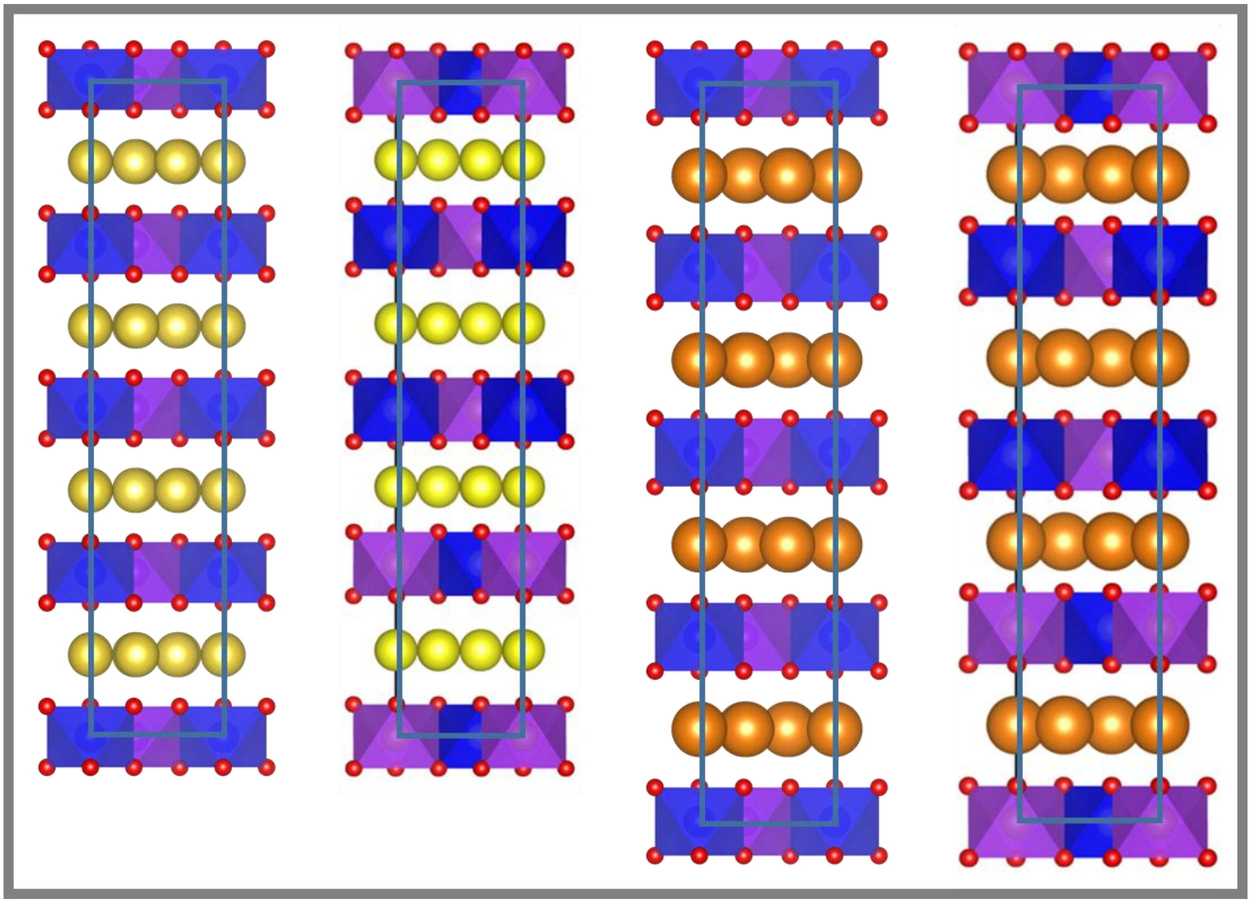

Supplementary Figure 33. Exemplar configurations of $Na_2Ni_2TeO_6$ and $K_2Ni_2TeO_6$ parent phase compositions (some with varying shifts of the Te/Ni slabs where Na atoms reside) assessed using theoretical computation. Their formation energies were calculated based on DFT calculations.

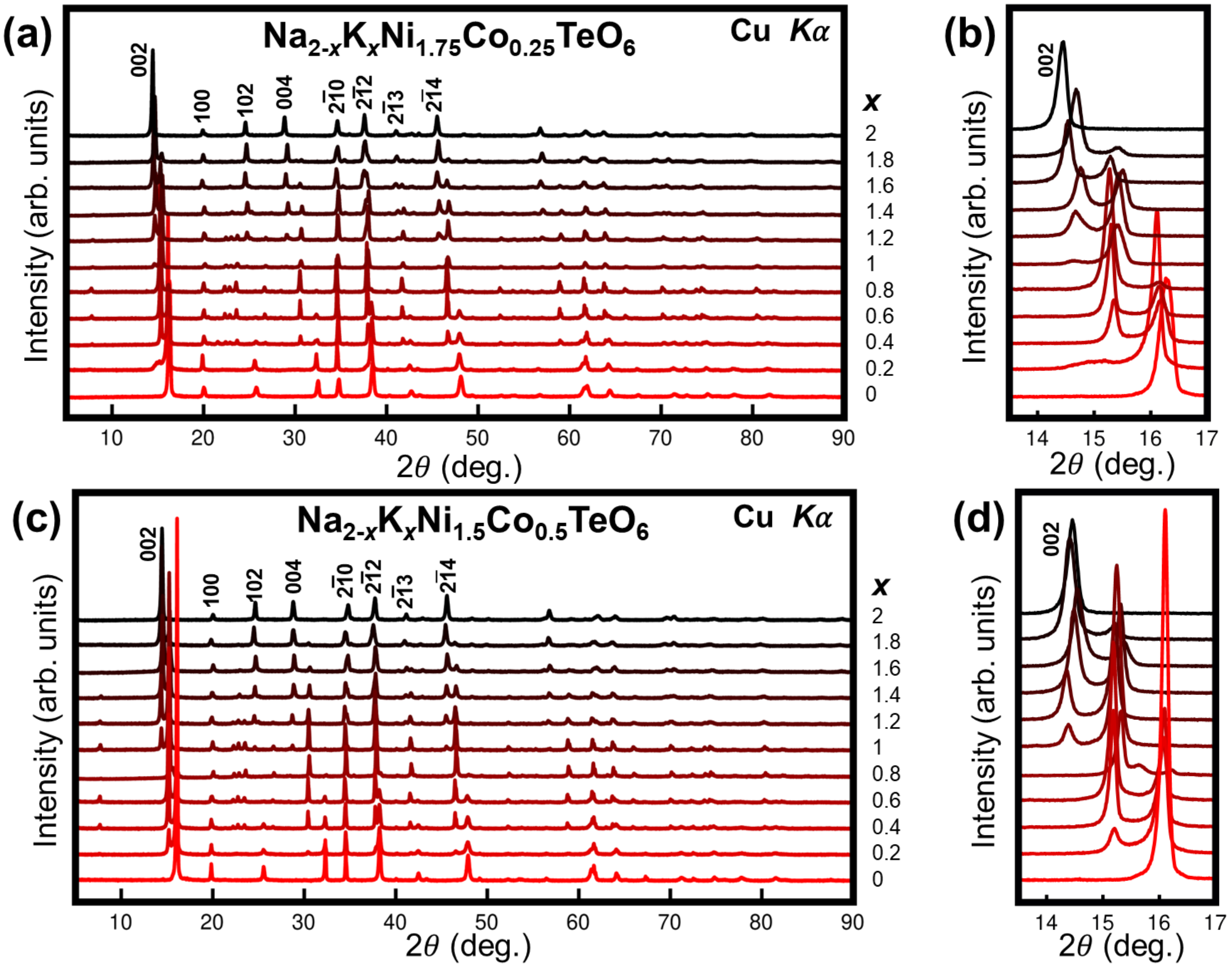

Supplementary Figure 34. (a) X-ray diffraction (XRD) patterns of $Na_{2-x}K_xNi_{1.75}Co_{0.25}TeO_6$ ($0 \leq x \leq 2$) and **(b)** Evolution of the (002) Bragg peaks with varying amounts of Na and K content (x). **(c)** XRD patterns of $Na_{2-x}K_xNi_{1.5}Co_{0.5}TeO_6$ and **(d)** An enlarged image showing the evolution of (002) Bragg peaks. Bragg peaks for $K_2Ni_{1.75}Co_{0.25}TeO_6$ (indexed in the $P6_3/mcm$ hexagonal space group) are shown in black.

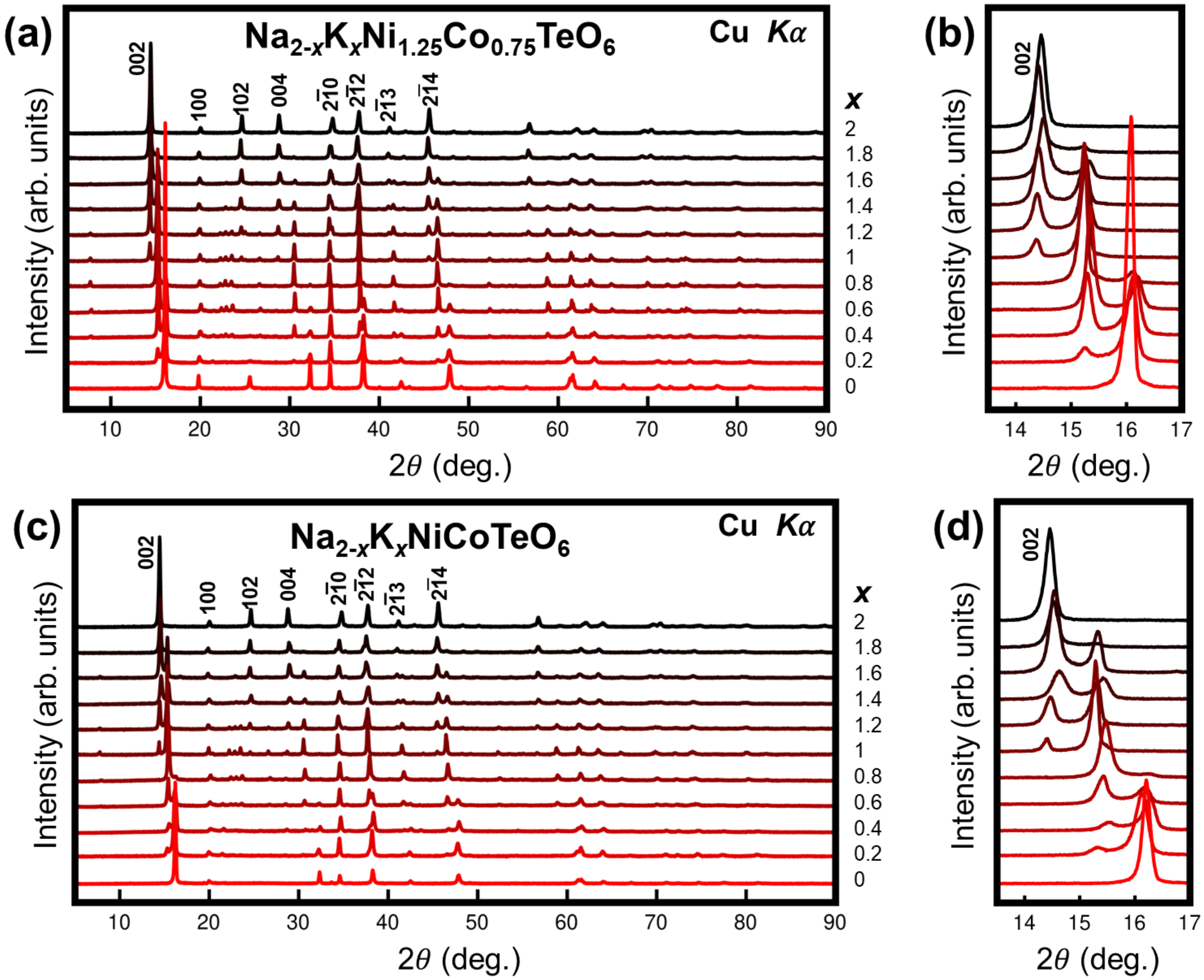

Supplementary Figure 35. (a) X-ray diffraction (XRD) patterns of $Na_{2-x}K_xNi_{1.75}Co_{0.25}TeO_6$ ($0 \leq x \leq 2$) and **(b)** Evolution of the (002) Bragg peaks with varying amounts of Na and K content (x). **(c)** XRD patterns of $Na_{2-x}K_xNi_{1.5}Co_{0.5}TeO_6$ and **(d)** An enlarged image showing the evolution of (002) Bragg peaks. Bragg peaks for $K_2Ni_{1.75}Co_{0.25}TeO_6$ (indexed in the $P6_3/mcm$ hexagonal space group) are shown in black.

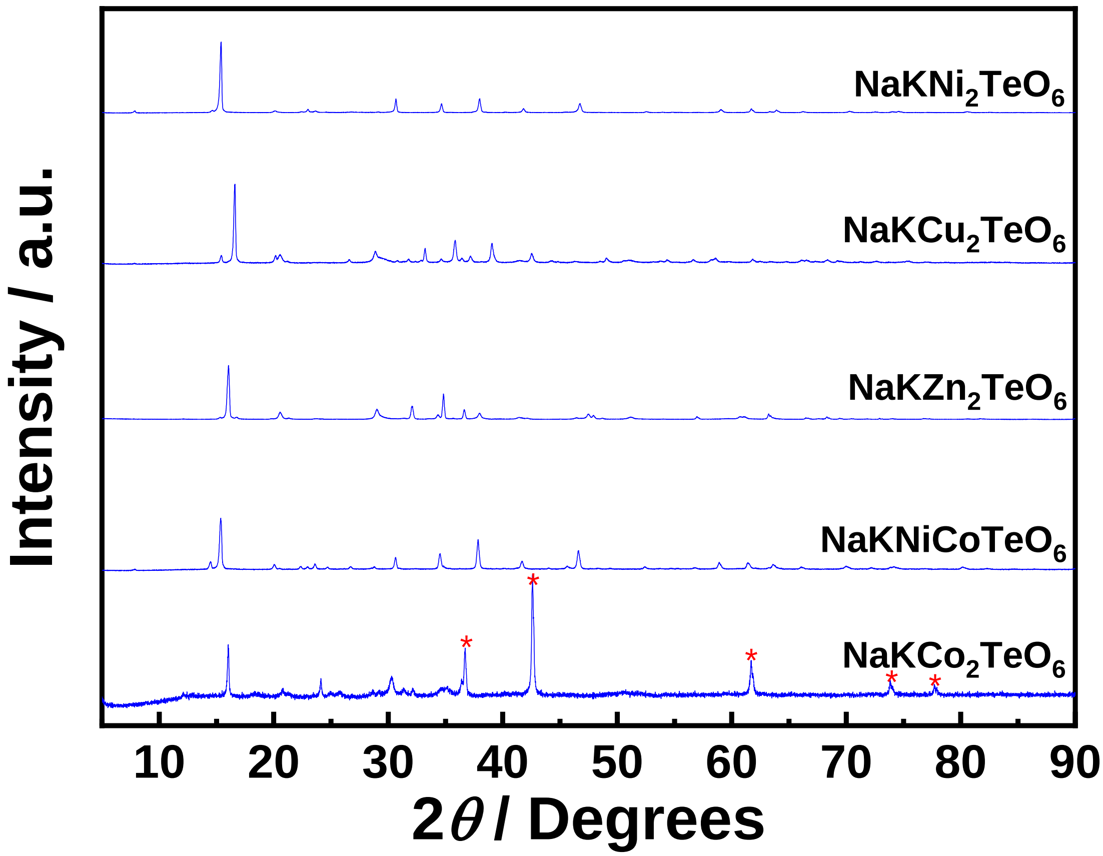

Supplementary Figure 36. XRD patterns of $Na_2KM_2TeO_6$ (M = Cu, Zn and Co) showing the possibility to design related mixed alkali compositions as $NaKNi_2TeO_6$. CoO impurity peaks that were traced in the diffraction pattern of $NaKCo_2TeO_6$ are shown in asterisks.

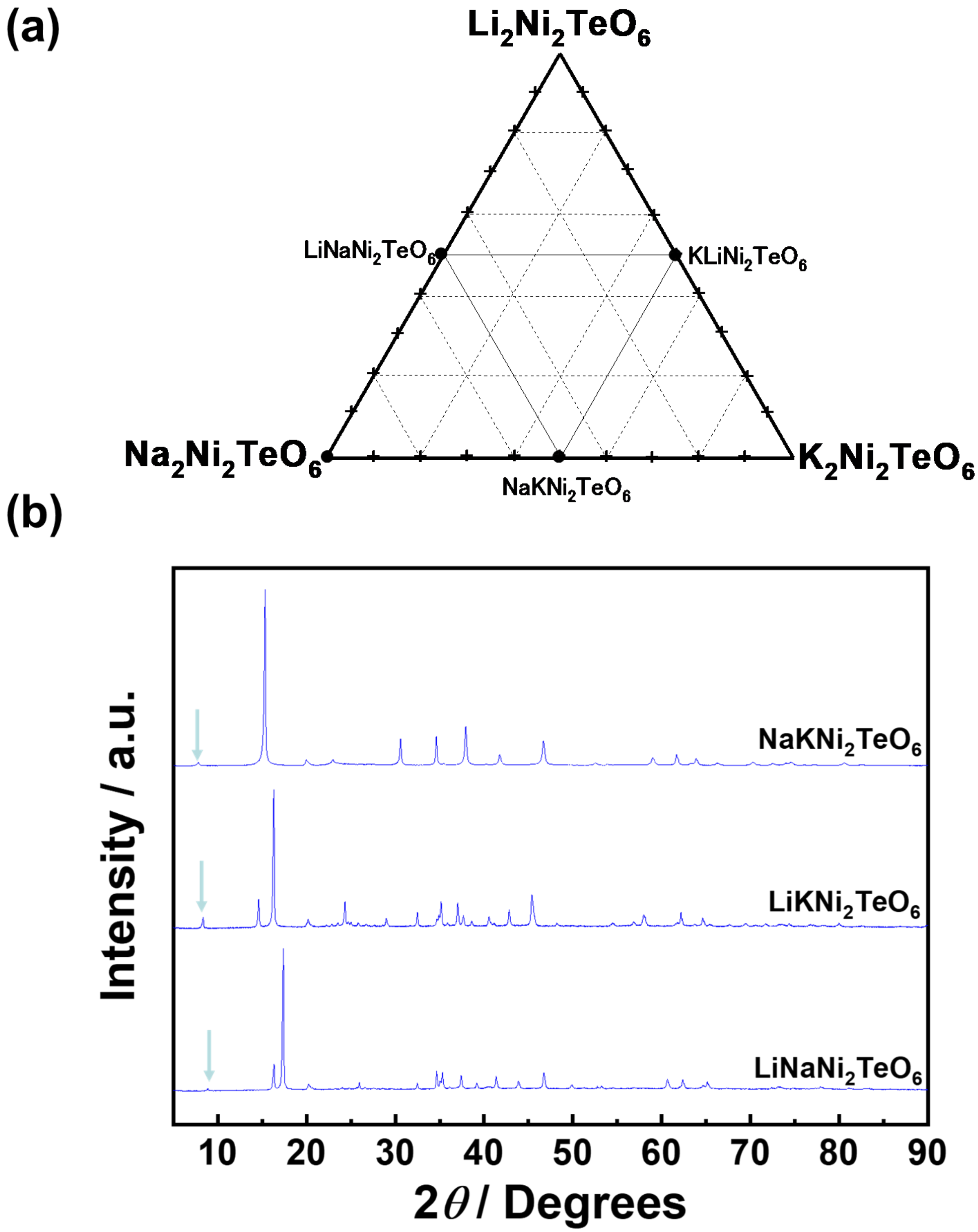

Supplementary Figure 37. (a) $Li_2Ni_2TeO_6$-$Na_2Ni_2TeO_6$-$K_2Ni_2TeO_6$ phase diagram showing new mixed alkali compositions. **(b)** XRD patterns of other conceivable mixed alkali honeycomb layered oxides synthesised in the course of the study. Bragg peak at low diffraction angle that is reminiscent of the mixed-alkali ordering is shown in arrow.

Supplementary Table 1. Summary of the quantitative elemental composition of prime intermediate $Na_{2-x}K_xNi_2TeO_6$ (x = 0, 0.2, 0.6, 1.0, 1.4, 1.8 and 2.0) compositions of mixed-alkali honeycomb layered oxides.

	atomic weight %				element ratio			
	Na	K	Ni	Te	Na	K	Ni	Te
$Na_2Ni_2TeO_6$	34.72	0.00	44.40	20.88	1.7	0.0	2.1	1.0
$Na_{1.8}K_{0.2}Ni_2TeO_6$	36.53	4.45	37.76	21.27	1.7	0.2	1.8	1.0
$Na_{1.4}K_{0.6}Ni_2TeO_6$	27.58	10.15	41.94	20.34	1.4	0.5	2.1	1.0
$NaKNi_2TeO_6$	18.98	18.00	42.41	20.60	0.9	0.9	2.1	1.0
$Na_{0.6}K_{1.4}Ni_2TeO_6$	11.55	26.73	40.81	20.91	0.6	1.3	2.0	1.0
$Na_{0.2}K_{1.8}Ni_2TeO_6$	4.03	33.89	41.73	20.35	0.2	1.7	2.1	1.0
$K_2Ni_2TeO_6$	0.48	38.12	41.37	20.03	0.0	1.9	2.1	1.0

We noted a propensity to prepare highly stoichiometric compositions using slightly an excess amount of alkali carbonates.

Supplementary Table 2. Composition stoichiometry of constituent Na, K, Ni and Te in pristine $NaKNi_2TeO_6$. Table showing the experimental stoichiometry values obtained from inductively coupled plasma atomic emission spectroscopy (ICP-AES) measurement of pristine $NaKNi_2TeO_6$ synthesised at 800°C in this study.

compound	Na (mole ratio)	K (mole ratio)	Ni (mole ratio)	Te (mole ratio)
$NaKNi_2TeO_6$	0.999($\mp$0.001)	0.995($\mp$0.005)	2.01($\mp$0.01)	1

Supplementary Table 3. Structural parameters obtained from the Rietveld refinement of the synchrotron XRD pattern of $NaKNi_2TeO_6$ indexed in the $P\bar{6}2c$ hexagonal space group. Atomic coordinates (*x, y, z*), occupancies (*g*), and isotropic atomic displacement parameters (U_{iso}) obtained by Rietveld refinement of synchrotron X-ray diffraction data for as-prepared $NaKNi_2TeO_6$ indexed in the space group $P\bar{6}2c$ (hexagonal) with lattice constants *a* = 5.2252(1) Å, *c* = 23.561(1) Å (R_{wp} = 13.84 %, R_p = 9.57 %, GOF (χ^2) = 6.37). The isotropic thermal factors (U_{iso}) of the K1, K2, K3 and K4 sites were constrained. The U_{iso} of Ni1 and Ni2 were constrained to 0.003 Å^2. Further details of the crystal structure can be obtained from the Fachinformationszentrum Karlsruhe, D-76344 Eggenstein-Leopoldshafen (Germany), on quoting the depository (accession) numbers CSD-2070815.

Atom	*g*	*x*	*y*	*z*	U_{iso}
**Te1**	1	2/3	1/3	0.11848(11)	0.0077(4)
**Ni1**	1	0	0	0.1193(3)	0.003
**Ni2**	1	1/3	2/3	0.11681(18)	0.003
**K1**	1/3	0	0	1/4	0.105(7)
**K2**	1/3	2/3	0	1/4	0.105(7)
**K3**	1/3	2/3	1/3	1/4	0.105(7)
**K4**	1/3	1/3	2/3	1/4	0.105(7)
**Na1**	2/3	1/3	0	0	0.046(5)
**O1**	1	1/3	1/3	0.0689(5)	0.033(3)
**O2**	1	1/3	0	0.1602(3)	0.0003(14)

Supplementary Table 4. Structural parameters obtained from the Rietveld refinement of the neutron diffraction pattern of $NaKNi_2TeO_6$ indexed in the $P\bar{6}2c$ hexagonal space group with lattice parameters: a = 5.227237(8) Å, c = 23.58788(6) Å (R_{wp} = 10.76 %, R_p = 8.92 %, GOF (χ^2) = 6.78). Note that the z coordinate of the Te1, Ni1, and Ni2 were constrained. The isotropic thermal factors (B_{iso}) of all cations were constrained. The B_{iso} values of O1 and O2 were also constrained.

Atom	Occupancy	x	y	z	B_{iso} (Å^2)
**Te1**	1	2/3	1/3	0.117611(7)	0.231(3)
**Ni1**	1	0	0	0.117611(7)	0.231(3)
**Ni2**	1	1/3	2/3	0.117611(7)	0.231(3)
**K1**	1/3	0	0	1/4	0.231(3)
**K2**	1/3	2/3	0	1/4	0.231(3)
**K3**	1/3	2/3	1/3	1/4	0.231(3)
**K4**	1/3	1/3	2/3	1/4	0.231(3)
**Na1**	2/3	1/3	0	0	0.231(3)
**O1**	1	1/3	1/3	0.071957(13)	0.614(3)
**O2**	1	1/3	0	0.161895(13)	0.614(3)

Supplementary Table 5. Inter atomic pair potential parameters in equation 1 (refer to main manuscript) employed in the present study.[b]

x	q_x (C)	σ_x (Å)	A_{NaX} (eV)	A_{KX} (eV)	A_{XO} (eV)	C_{XO} (eVÅ^6)	P_{XO} (eV)	n_{XO}
Na	0.65	1.13	2.298	0.000[a]	0.096[a]	0.00	0.00	9
K	0.65	1.38	0.000[a]	0.015[a]	0.068[a]	0.00	0.00	9
Ni	1.30	0.74	24.256[a]	4.528[a]	3.085	59.85	31.00	9
Te	3.90	0.70	9.081	2.220	3.910	17.15	11.30	9
O	-1.30	1.21	0.096	0.068	0.926	85.14	0.00	7

$n_{X\text{-}X}$ = 11 (X = Na, K, Ni, and Te)

[a]The parameters are different than the reported.[1]
[b]Any parameters not listed in the table are assumed to be zero.

Supplementary Table 6. Comparison of simulated box dimensions calculated from NPT-MD simulation (300 K and 0 GPa pressure) and X-ray diffraction results.

Simulation Box Dimension	$7a$ (Å)	$7b$ (Å)	c (Å)
Experiment	36.85	36.85	23.50
MD (Δ(%))	37.90 (2.9)	37.90 (2.9)	24.18 (2.9)

Δ(%) = deviation from experimental box dimensions.

Supplementary Table 7. Electrochemical properties of positive electrode (cathode) materials reported for NaK anodes.
[a] Theoretical capacity calculated assuming full (de)intercalation of alkali cations.

Positive electrode material (wt.% of the active material in composite)	Negative electrode material	Electrolyte	Average voltage / V	Theoretical capacity [a] / mAh g^{-1}	Specific discharge capacity / mAh g^{-1}	Remarks	Ref.	Coulombic efficiency & Temperature
$NaKNi_2TeO_6$ (70%)	NaK	0.5 M NaFSI + 0.5 M KFSI in $Pyrr_{13}FSI$	3.8	133	82	Layered structured ($P6_3/mcm$)<br>First inorganic stable host containing both Na and K<br>Highest average voltage	This work	98% Room temperature
	Na	1.0 M NaFSI in $Pyrr_{13}FSI$	3.8		86			
	K	1.0 M KFSI in $Pyrr_{13}FSI$	3.9		52			
$Na_2MnFe(CN)_6$ (70%)	NaK	1.0 M $NaClO_4$ in PC	3.4	171	~120	Prussian blue analogue with preference to K ions (transformed to $K_2MnFe(CN)_6$ after few cycles)	**2**	99.64% Room temperature
	NaK	1.0 M $KClO_4$ in PC	3.5		~125			97~99% Room temperature
$K_{0.220}Fe[Fe(CN)_6]_{0.805}$ (80%)	NaK	0.8 M KPF_6 in EC/DEC (1:1 vol%)	3.2	114	107	Prussian blue analogue as host for K ions	**3**	~93% Room temperature
$Na_2C_6O_6$ (70%)	NaK	1.0 M $NaClO_4$ in EC/DEC (1:1 vol%) with 10 vol% FEC	2.2	250	~130	Carbonyl-based organic host with negligible preference toward Na and K	**4**	100%
Poly(hexaaza-trinapthylene) (40%)	NaK	1.5 M KPF_6 in DME	1.8	--	~180	Organic polymer host with preference to K ions	**5**	~100% Room temperature
TiO_2 embedded NPCTO (70%)	NaK	1.0 M $NaClO_4$ in EC/DEC (1:1 vol%) with 5 wt% FEC	1.0	--	~300	Capacitive capacity dominated	**6**	~100% Room temperature
Ga-In (% not disclosed)	NaK	1.0 M $NaClO_4$ in DME/FEC (95:5 vol%)	0.5	--	~80	Liquid alloy host with preference to Na ions	**7**	~100% Room temperature

Abbreviations: FSI: bis(fluorosulphonyl)imide; $Pyrr_{13}$: *N*-methyl-*N*-propylpyrrolidinium; PC: propylene carbonate; EC: ethyl carbonate; DEC: diethyl carbonate; FEC: fluoroethylene carbonate; DME: 1,2-dimethoxyethane; NPCTO: nitrogen-doped porous carbon truncated octahedra sheets

Supplementary Table 8. Physicochemical properties of a mixed ionic liquid comprising a 0.5 M NaFSI + 0.5 M KFSI in $Pyr_{13}FSI$ ($Na_{0.10}K_{0.10}Pyr_{0.80}FSI$). Data for 1 M KFSI in $Pyr_{13}FSI$ ($K_{0.20}Pyr_{0.80}FSI$) and 1 M NaFSI in $Pyr_{13}FSI$ ($Na_{0.20}Pyr_{0.80}FSI$) ionic liquid electrolytes have been provided for a comparison. Measurements were performed at room temperature (25°C). For clarity, 'M' here denotes 'mol dm^{-3}'.

Ionic liquids	Density / g cm^{-3}	Viscosity / mPa s	Ionic conductivity / mS cm^{-1}
1.0 M KFSI/$Pyr_{13}FSI$	1.4226	83.9	4.4
1.0 M NaFSI/$Pyr_{13}FSI$	1.4119	94.9	4.1
0.5 M NaFSI + 0.5 M KFSI/ $Pyr_{13}FSI$	1.4232	89.2	4.3

Supplementary Note 1. Shearing of the adjacent Te/Ni slabs where Na atoms reside.

Further nanostructural details relating to the Na-rich phases reveal stacking disorders along the *c*-axis, as is schematically shown in **Figure R1**, entailing shifts/shear of the transition metal slabs along the *ab* plane by a Burgers vector corresponding to [±2/3 ±1/3 0].

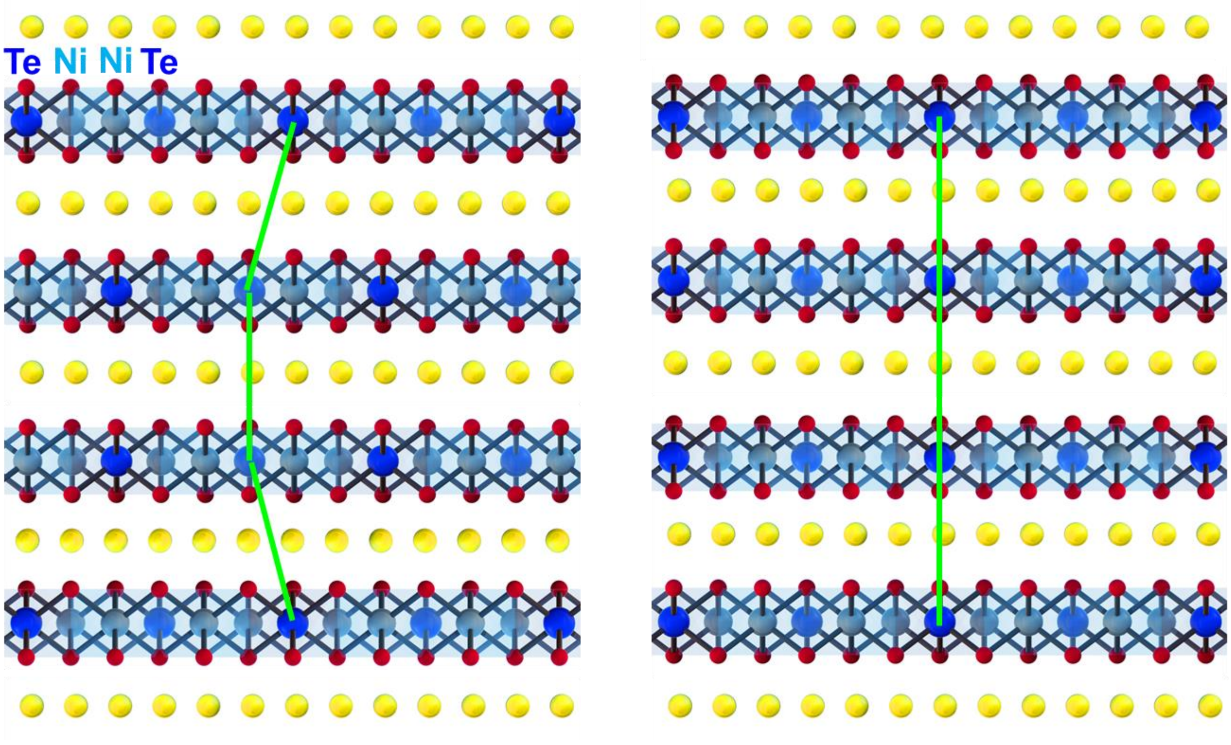

Figure R1. Stacking arrangement of the Na-rich phase $Na_2Ni_2TeO_6$ attained upon subsequent cycling of $NaKNi_2TeO_6$ in NaK half-cells.

The slabs in the Na-rich phase ($Na_2Ni_2TeO_6$) are observed to deviate from the vertical arrays in certain domains (as highlighted by the green lines in **Figure R1**). For the sake of rigour, a shear transformation is defined by matrices of the type,

$$S_a = \begin{pmatrix} 1 & 0 & \lambda_a \\ 0 & 1 & 0 \\ 0 & 0 & 1 \end{pmatrix}, S_b = \begin{pmatrix} 1 & 0 & 0 \\ 0 & 1 & \lambda_b \\ 0 & 0 & 1 \end{pmatrix},$$

where S_a and S_b are the shear matrices in the *a* and *b* directions respectively. Due to the differing crystalline positions of Ni atoms relative to Te atoms in the unit cell at the stacking faults, the Burgers vector corresponds to $\lambda_a = \pm 2/3$ and $\lambda_b = \pm 1/3$ anisotropic shear transformations along the

ab plane. Equivalently, the shear transformations lead to a Ni atom exchanging relative positions with a Te atom within the unit cell. This requires that the unit basis vector perpendicular to the *ab* plane (pointing in the *c* axis) given by the transpose of the basis vector [0, 0, 1] be multiplied by the combined transformation matrix $S = S_a S_b = S_b S_a$ given by,

$$S = \begin{pmatrix} 1 & 0 & \lambda_a \\ 0 & 1 & \lambda_b \\ 0 & 0 & 1 \end{pmatrix},$$

to yield, [±2/3, ±1/3, 1] but leaves unchanged the transpose basis vectors in the *a*- and *b*-axes *i.e.* [1, 0, 0] and [0, 1, 0] vectors respectively. Correspondingly, the Burgers vector [±2/3 ±1/3 0] is the difference between the transformed basis vector [±2/3, ±1/3, 1] and the original unit basis vector [0, 0, 1] as shown in the **Rendition R1** below:

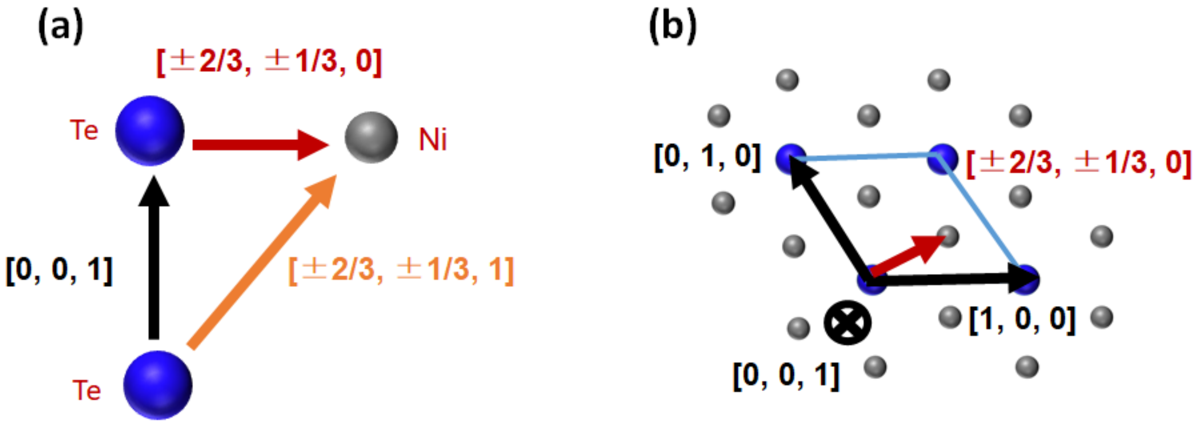

Rendition R1: The basis vectors defined on the unit cell relative to the shear transformation. (a) The *c*-zone axis unit basis vector [0, 0, 1], its transformed vector [±2/3, ±1/3, 1] under shear transformation, *S* and the corresponding Burgers vector [±2/3, ±1/3, 0] depicting the Te ion occupying the relative position of Ni. (b) The shear transformation in (a) as seen from the [001] axis (*ab* plane) showing the unit basis vectors [0, 0, 1], [0, 1, 0] and [1, 0, 0], and the Burgers vector, [±2/3, ±1/3, 0].

Supplementary References

1. Sau, K. & Kumar, P. P. Ion Transport in $Na_2M_2TeO_6$: Insights from Molecular Dynamics Simulation. *J. Phys. Chem. C* **119** 1651–1658 (2015).
2. Xue, L. G., Gao, H. C., Li, Y. T. & Goodenough, J. B. Cathode dependence of liquid-alloy Na-K anodes. *J. Am. Chem. Soc.* **140**, 3292–3298 (2018).
3. Zhang, L. Y., Xia, X. H., Zhong, Y., Xie, D., Liu, S. F., Wang, X. L. & Tu, J. P. Exploring self-healing liquid Na-K alloy for dendrite-free electrochemical energy storage. *Adv. Mater.* **30**, 1804011 (2018).
4. Ding, Y., Guo, X. L. Qian, Y. M. Zhang, L. Y. Xue, L. G., Goodenough, J. B. & Yu, G. H. A liquid-metal-enabled versatile organic alkali-ion battery. *Adv. Mater.* **31**, 1806956 (2019).
5. Kapaev, R. R., Obrezkov, F. A., Stevenson, K. J. & Troshin, P. A. Metal-ion batteries meet supercapacitors: high capacity and high rate capability rechargeable batteries with organic cathode and a Na/K alloy anode. *Chem. Commun.* **55**, 11758–11761 (2019).
6. Huang, M., Xi, B. J., Feng, Z. Y., Wu, F. F., Wei, D. H., Liu, J. Feng, J. K., Qian, Y. T. & Xiong, S. L. New insights into the electrochemistry superiority of liquid Na-K alloy in metal batteries. *Small* **15**, 1804916 (2019).
7. Ding, Y., Guo, X. L., Qian, Y. M., Xue, L. G., Dolocan, A. & Yu, G. H. Room-temperature all-liquid-metal batteries based on fusible alloys with regulated interfacial chemistry and wetting. *Adv. Mater.* **32**, 2002577 (2020).
