# Supplementary material for: Mixed alkali-ion transport and storage in atomic-disordered honeycomb layered NaKNi2TeO6
Source: Nat Commun. 2021 Aug 2;12:4660. doi: 10.1038/s41467-021-24694-5 (PMC8329229; doi:10.1038/s41467-021-24694-5)
Supplement: Supplementary file 3 — Description of Additional Supplementary Information [file 41467_2021_24694_MOESM3_ESM.pdf]

Molecular dynamic simulation of $NaKNi_2TeO_6$ at 600K, showing the dynamic behaviour of Na^+ and K^+ atoms.
